# Structural Properties of Metal–Organic Frameworks at Elevated Thermal Conditions via a Combined Density Functional Tight Binding Molecular Dynamics (DFTB MD) Approach

**DOI:** 10.1021/acs.jpcc.2c05103

**Published:** 2023-01-10

**Authors:** Felix R. S. Purtscher, Leo Christanell, Moritz Schulte, Stefan Seiwald, Markus Rödl, Isabell Ober, Leah K. Maruschka, Hassan Khoder, Heidi A. Schwartz, El-Eulmi Bendeif, Thomas S. Hofer

**Affiliations:** † Institute of General, Inorganic, and Theoretical Chemistry, Center for Chemistry and Biomedicine, University of Innsbruck, Innrain 80-82, A-6020Innsbruck, Austria; ‡ CRM2 UMR, CNRS 7036, Université de Lorraine, F-54000Vandæuvre-lès-Nancy, France

## Abstract

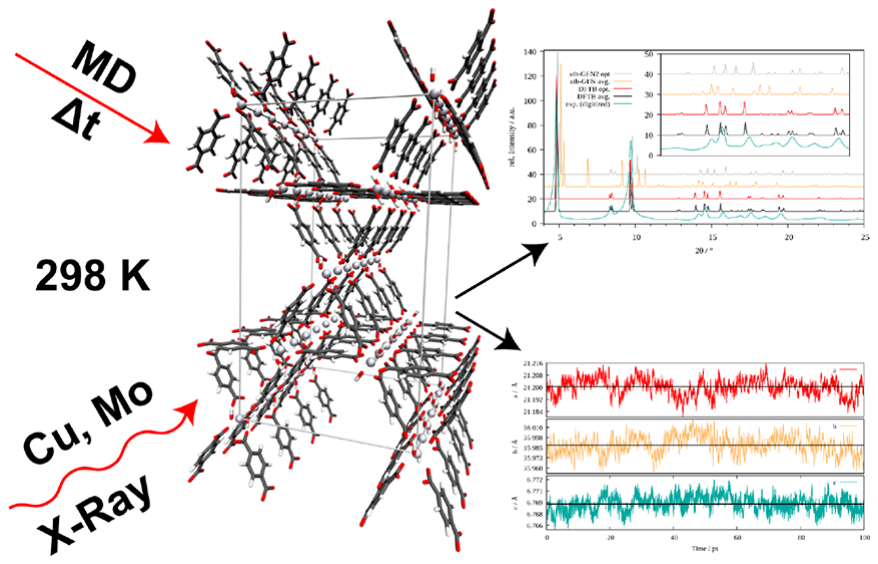

The performance of different density functional tight binding (DFTB) methods for the description of six increasingly complex metal–organic framework (MOF) compounds have been assessed. In particular the self-consistent charge density functional tight binding (SCC DFTB) approach utilizing the 3ob and matsci parameter sets have been considered for a set of four Zn-based and two Al-based MOF systems. Moreover, the extended tight binding for geometries, frequencies, and noncovalent interactions (GFN2-xTB) approach has been considered as well. In addition to the application of energy minimizations of the respective unit cells, molecular dynamics (MD) simulations at constant temperature and pressure conditions (298.15 K, 1.013 bar) have been carried out to assess the performance of the different DFTB methods at nonzero thermal conditions. In order to obtain the XRD patterns from the MD simulations, a flexible workflow to obtain time-averaged XRD patterns from (in this study 5000) individual snapshots taken at regular intervals over the simulation trajectory has been applied. In addition, the comparison of pair-distribution functions (PDFs) directly accessible from the simulation data shows very good agreement with experimental reference data obtained via measurements employing synchrotron radiation in case of MOF-5. The comparison of the lattice constants and the associated X-ray diffraction (XRD) patterns with the experimental reference data demonstrate, that the SCC DFTB approach provides a highly efficient and accurate description of the target systems.

## 1 Introduction

Since their emergence in the late 1990s metal–organic frameworks^1^ (MOFs) established a new area of research focusing on the synthesis of new functional materials. Featuring manifold possibilities in their application while enabling straightforward and inexpensive strategies in their synthesis, MOF research became one of the most active areas in modern material sciences.^2−4^ The highly porous, crystalline structure of MOFs enables the trapping of small molecular compounds, resulting for instance in high gas storage capacities^5−7^ along with a potential activation of guest molecules via the incorporation of catalytically active reaction centers inside the host structure.^8−10^ More advanced applications focusing on (semi)-conducting properties,^11^ the synthesis of photoswitchable functional materials,^12,13^ and the exploitation of MOFs as carrier matrix in improved drug delivery systems^14,15^ have recently attracted increased attention.

As a consequence of their supramolecular nature MOFs typically feature large unit cells containing a considerable number of atoms including at least one type of metal ion.^1^ Since the theoretical description of interactions involving the latter are typically more challenging compared to (bio)organic molecules, classical molecular mechanical (MM) approaches^16,17^ are typically limited in the achievable accuracy. Classical force field (FF) have been applied with great success in modeling the properties of pristine MOF systems,^18,19^ e.g. the bulk moduli and linear thermal expansion coefficients.^20^ However, it was also noted that more intricate geometries (e.g., MOFs containing open-metal sites)^19^ are oftentimes not represented with quantitative accuracy.^18^ In addition, the properties of the MOF itself are oftentimes not of particular interest, but the interactions with guest molecules embedded inside the host structure. In this context, a number of studies^21−23^ have shown, that the achievable accuracy may be limited when employing a FF-based description. While these studies have indicated that polarizable FF approaches may improve the achievable accuracy, they are much more challenging in their parametrization and at the same time considerably more demanding in terms of execution.

Quantum mechanical (QM) calculation methods^24,25^ provide a suitable alternative, since effects arising from the (re)distribution of the electron density such as polarization, charge-transfer and many-body interactions are explicitly taken into account. While a large number of properties can be determined via the explicit treatment of the electron distribution (e.g., vibrational/phonon modes, electronic band structures, etc.), the associated computational demand is massively increased over the application of the conceptually much simpler MM methods. As a consequence, QM-based investigations are in many cases limited to a small number of representative configurations, typically determined via an energy minimization^16,17^ achieved by optimizing the positions of the nuclei and, in case of periodic systems, the parameters of the unit cell. The resulting structure thus corresponds to a minimum on the potential energy surface.

However, when disregarding the influence of quantum effects associated with the nucleic degrees of freedom as typically done within the framework of the Born–Oppenheimer approximation,^24,25^ investigations carried out at such a minimum configuration have to be considered as a 0 K structure. Although such a treatment may prove sufficient for a variety of research questions, the use of energy-minimized structures gives rise to a number of potential limitations in computational studies. If the employed theoretical level is not sufficiently accurate, then the structure of the MOF might undergo a phase transition or even collapse at elevated temperatures, implying that it is only artificially stabilized via the 0 K treatment. As such the underlying thermodynamical properties associated with the theoretical treatment cannot be unambiguously validated. Even if the theoretical level is adequate, the negative expansion coefficient typical for many MOF systems^26−28^ will inherently result in a deviation from experimentally determined properties measured at elevated temperatures. Finally, when investigating the interactions of guest molecules embedded in the MOF structure no equilibration of the system is possible, since the associated kinetics are effectively deactivated thus eliminating any relevant guest mobility. The latter implies that the guest molecule will orient toward the closest energy minimum with respect to the (oftentimes arbitrarily chosen) initial structure, even if this configuration is not relevant for the actual host–guest interaction adapted at experimental conditions.

Whenever such considerations become relevant in a particular investigation, the description of the systems at elevated temperature via a suitable ensemble-based approach such as Monte Carlo and molecular dynamics (MD) simulations^29−31^ provides a more adequate approach. However, the considerable computational demand of periodic quantum chemistry even when employing low-level implementations of density functional theory (DFT)^32,33^ at the generalized-gradient approximation (GGA) level may prove prohibitively expensive when applied within such a simulation framework. This limitation is further amplified by the presence of guest molecules, which can be expected to greatly reduce the symmetry of the combined guest@MOF system. This effectively prevents the exploitation of space group symmetry in the demanding QM calculation, which is often employed in calculations of highly ordered MOF structures.

A particularly promising alternative approach applied with large success also in the treatment of MOF systems are density functional tight binding (DFTB) approaches,^34−39^ providing a remarkable compromise between computational cost and quality of results. The derivation of DFTB methods is based on a Taylor series of the DFT Kohn–Sham energy expression with respect to the equilibrium density, which represents an entirely different paradigm as Hartree–Fock based semiempirical methods.^40,41^ However, similar to the latter DFTB-based methods are strongly dependent on an adequate derivation of the atomic interaction parameters. Typically, self-consistent charge density functional tight binding (SCC DFTB) is up to a factor of 100 faster than the respective parent DFT methods, while the obtained results are oftentimes of comparable accuracy if the system of interest remains within the scope of the employed parametrization.

Indeed, a number of studies have successfully applied DFTB-based calculations methods for the treatment of metal–organic frameworks. Heine and co-workers report good agreement of SCC DFTB calculations carried out for a variety of Zn-, Cu-, and Al-based MOFs against DFT calculation results and experimental reference data.^42^ Sohlberg et al. also report very good agreement of DFTB against DFT in a study of CO_2_ absorption in Zn·ADC (9,10-anthracenedicarboxylic acid).^43^ Similarly, Huang et al. report good agreement between theoretical predictions of H_2_O adsorption and desorption in Zn-MOF-74 with experimental data, while Allendorf and co-workers^44^ investigated energetic and charge transfer properties of donor–acceptor pairs confined in different MOF structures. The results of experimental investigations as well as electronic structure calculations of MOF-177 also resulted in excellent agreement of structural and band structure properties using only a simple gamma point setup in the optimization, followed by an increase in the system size to a 3·3·3 supercell when calculating the density of states.^45^

These studies provide a clear indication that a DFTB-based treatment provides an efficient and at the same time accurate representation of MOF and guest@MOF systems in good agreement with experimental data and more demanding DFT calculations. The majority of these conclusions is based on optimized structures obtained via energy minimization, oftentimes considering also the relaxation of the unit cell in addition to the optimization of the atomic positions. The combination of molecular dynamics (MD) and SCC DFTB on the other hand provides a well-suited methodical framework that enables theoretical investigations of metal–organic frameworks at predefined state points corresponding to nonzero thermal conditions. This enables an analysis of the thermal stability of the MOF subject to the DFTB description or might be of advantage whenever ligand mobility in the host structure is of particular interest.^12^ The work by Li and co-workers focused on water adsorption in MOF-74^46^ is one of the rare examples of the application of a combined DFTB MD investigations.

Due to the considerable size of the unit cell the periodic treatment of the system via Brillouin-zone (BZ) integration typically carried out in reciprocal space^47^ can be kept to a minimum. In order to identify a suitable compromise between the computational effort and the accuracy in the description, time-averaged powder X-ray powder diffraction (XRD) patterns determined from the simulation data^48^ can be directly compared to their experimental counterparts. In addition, the comparison of atomic pair distribution functions (PDFs) obtained via X-ray measurements^49^ provides a direct route to assess the performance of the theoretical calculations.

In this work six increasingly complex metal–organic frameworks have been investigated via extensive DFTB MD simulations: the Zn-based metal–organic framework MOF-5^50^ known since 1999 employs 1,4-benzenedicarboxylate (BDC^2–^) as linker and is regarded as one of the most prominent and widely investigated examples of this material class. The more complex DMOF-1^51^ is a mixed-linker MOF employing in addition to BDC^2–^ diazabicyclo[2.2.2]octane (dabco) as linking unit. The ZIF-8 system^52^ represents one of the most widely investigated variant of the zeolitic imidazolate frameworks and therefore, has also been considered in this study. Porpyhrine-based MOFs^53^ have attracted increased attention due to their prospective catalytic activity and the performance of the outlined simulation methodology to the Zn-based robust porphyrinic material (RPM) framework^60^ was evaluated in this study as well. More specifically, the ZnZn-RPM system has been considered in this study, implying that both porphyrin units present in the unit cell are occupied by Zn^2+^-ions. Finally, MIL-68^54^ and MIL-53^55,56^ were chosen as examples of an MOF employing a trivalent ionic species in the secondary building unit (SBU), which in this case was Al^3+^.

In addition to probing the influence of nonzero thermal conditions on the description of these MOF systems, the impact associated with a reduced BZ sampling of the periodic system was of particular interest. Since the supramolecular nature of MOFs is associated with comparably large sizes of the associated unit cells, the computational effort associated with the sampling of the BZ carried out in reciprocal space can typically be kept to a minimum, until only the so-called Γ-point (i.e., the center of the BZ) is considered.

Moreover, DFTB MD simulation results obtained from MIL-68(Ga) are presented as an example of a nonideal case. As a consequence of the parametrization initially focused on Ga-containing semiconducting properties, the description of the MOF suffers from notable inaccuracies, foremost the structural collapse in simulations subject to external pressure. The possibility of constant volume simulations as a potential alternative strategy is outlined.

## 2 Methodology

**Figure 1:**
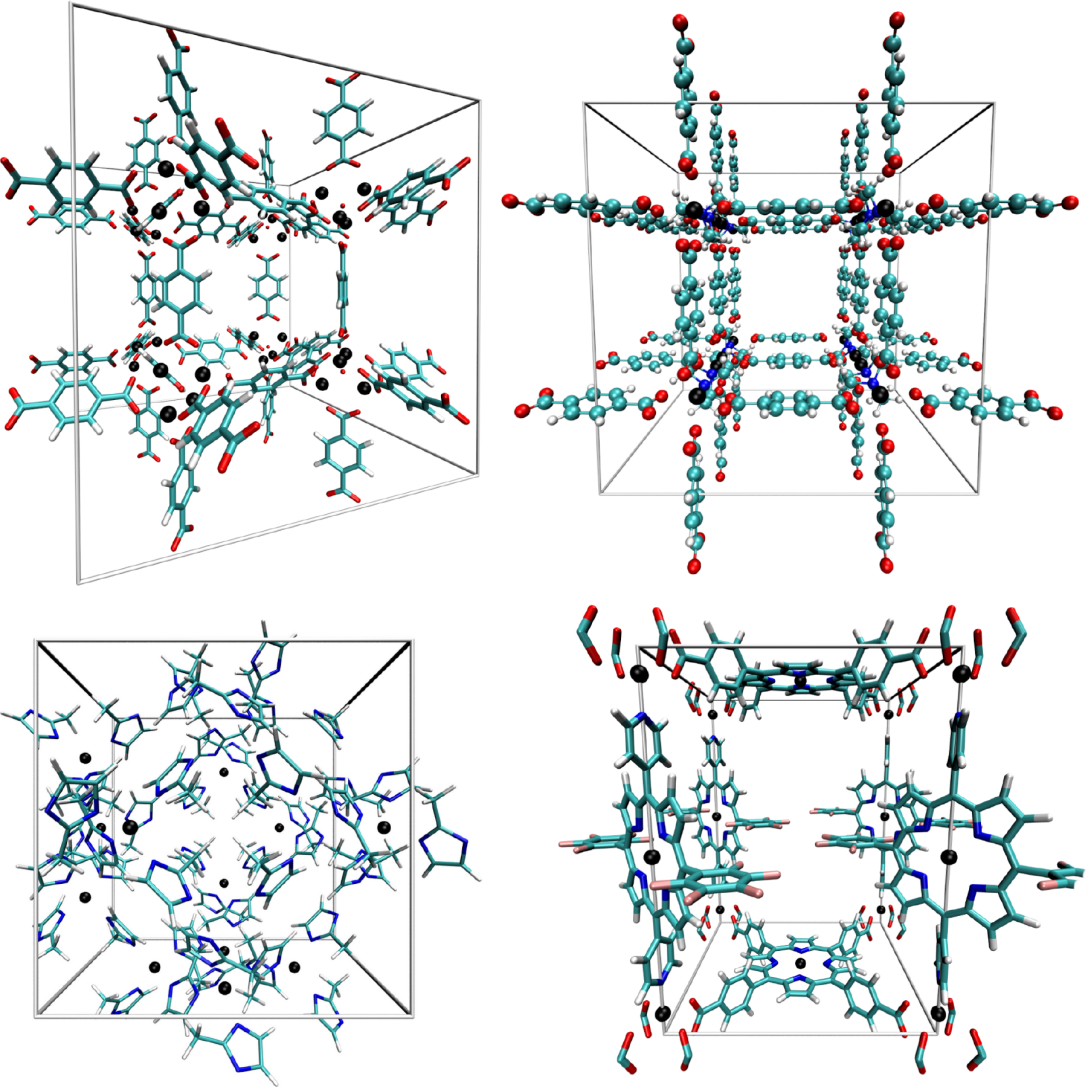
Screenshots depicting the unit cells employed in the simulation studies of MOF-5 (top left), DMOF-1 (top right), ZIF-8 (bottom left), and ZnZn-RPM (bottom right) . Atom colors: H - white, C - cyan, O - red, N - blue, Zn - black.

**Figure 2:**
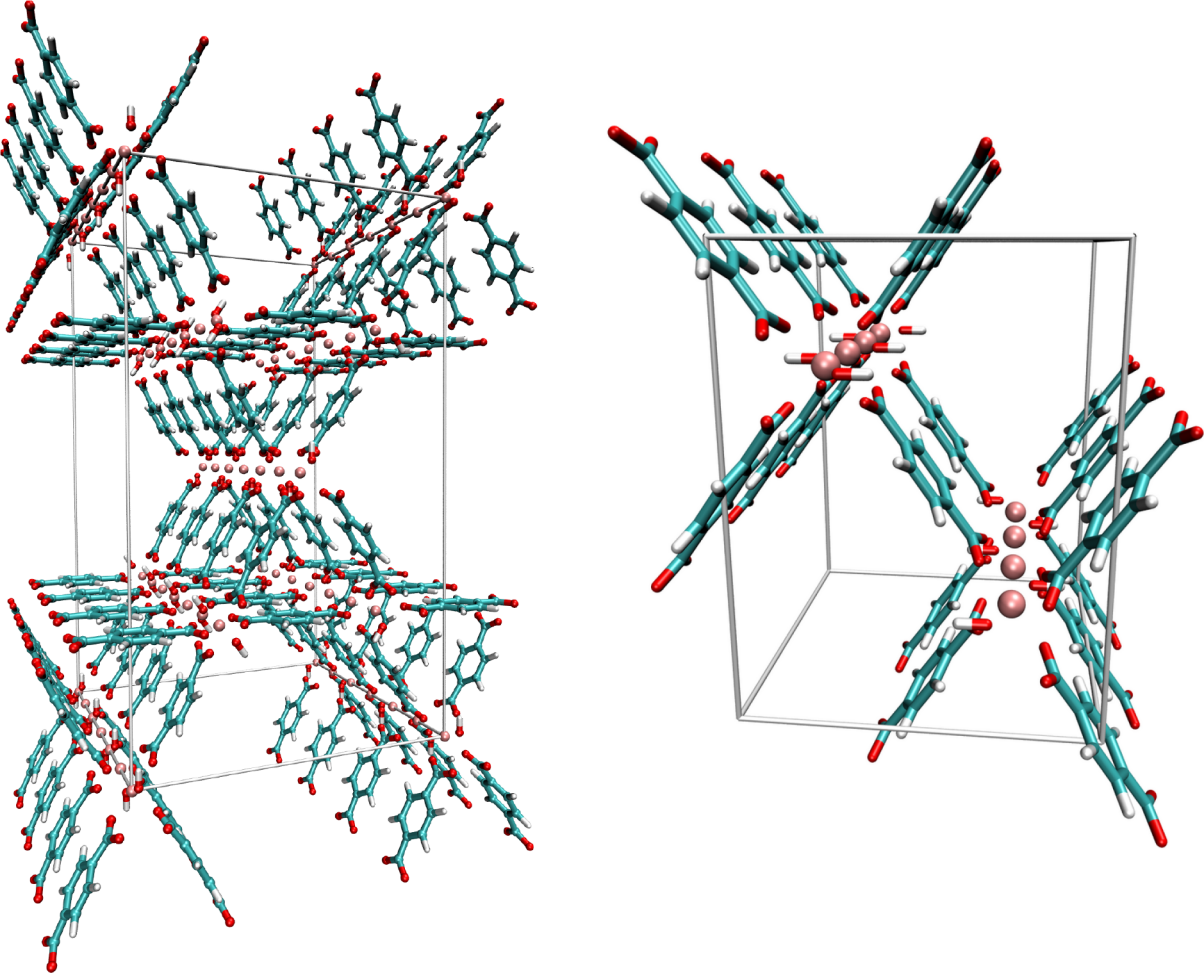
Screenshots depicting the unit cells employed in the simulation studies of MIL-68(Al) (left) and MIL-53(Al) (right). Atom colors: H - white, C - cyan, O - red, N - blue, Al - rose.

In the following, the strategy to calculate MD-averaged X-ray diffraction patterns and pair-distribution functions are outlined. Details with respect to the experimental procedure as well as the molecular dynamics simulations are provided in the Supporting Information, sections S1 and S2. Depictions of the equilibrated simulation systems are provided in Figures 1 and 2, respectively. The associated system sizes along with the atomic compositions and the employed radiation sources in the X-ray diffraction measurements are listed in Table 1.

### 2.1 Calculation of Powder X-ray Diffraction Patterns

In order to compare the structural description of the systems against experimental reference data, powder X-ray diffraction (PXRDs) patterns have been calculated using the RIETAN-FP suite^48^ included in the VESTA program.^57^ In addition to the diffractograms obtained for the minimized geometries of the individual MOFs (thereby also considering the relaxation of the cell parameters), time-averaged patterns have been evaluated from the SCC DFTB MD trajectory. In the latter case, every fifth configuration of the sampled trajectory was extracted and converted into a corresponding crystallographic information file (CIF). The latter were then employed as input into VESTA/RIETAN-FP to evaluate the associated PXRD data.

**Table 1:** Supercell Size, Chemical Formula, Number of atoms *N*_*atoms*_, and Valence Electrons *n*_*val*_ of the Investigated Systems and Wave Lengths of X-ray Radiation Employed in the Experimental XRD Measurements

system	cell size	formula	*N*_*Atoms*_	*n*_*val*_	source	λ/nm
MOF-5	1·1·1	C_192_ H_96_ O_104_ Zn_32_	424	1872	Mo K_α_	0.709319
DMOF-1	2·2·2	C_176_ H_160_ N_16_ O_64_ Zn_16_	432	1488	Mo K_α_	0.709319
ZIF-8	1·1·1	Zn_12_ C_96_ H_120_ N_48_	276	888	Mo K_α_	0.709319
Zn-RPM	1·1·1	Zn_10_ C_90_ H_40_ N_10_ O_8_ F_10_	162	616	Cu K_α_	1.540598
MIL-68(Al)	1·1·3	Al_36_ C_288_ H_180_ O_180_	684	2520	Cu K_α_	1.540598
MIL-53(Al)	2·1·1	Al_8_ C_64_ H_40_ O_40_	152	560	Cu K_α_	1.540598
MIL-68(Ga)	1·1·3	Ga_36_ C_288_ H_180_ O_180_	684	2520	Cu K_α_	1.540598

By employing the command line interface of VESTA^57^ the entire process was fully automated. Since the individual diffractograms only show reflections in the form of discrete lines, a Gaussian-based weighted kernel density estimation^58^ to broaden the individual reflexes was applied. The respective Gaussian factor was set to 1/25.0° resulting in an improved resemblance of experimental PXRD data. For visualization and comparison purposes, the most intense reflection of each diffractogram was employed to normalize the intensity to 100. For each system a total of 5000 individual PXRD patterns for structures extracted in equal intervals over the simulation trajectory were calculated and subsequently averaged.

### 2.2 Total Scattering Measurements and Pair Distribution Functions Analysis

Atomic PDFs provide an alternative representation of the total scattered X-ray intensities, which describe the atomic arrangements in real space without any crystallographic assumption. It may be simply seen as a radial distribution of all the interatomic distances *r*_*ij*_ in the considered sample. Atomic PDFs *G*(*r*) can also be calculated from the simulation trajectory as
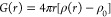
1with ρ_0_ being the corresponding atomic number density. The atomic pair density ρ(*r*) corresponds to the mean weighted density of neighboring atoms at a given distance *r* from an atom placed at the origin:^49^
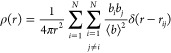
2with *b*_*i*_ an *b*_*j*_ being the scattering factors of the respective atoms and ⟨...⟩ denoting the associated ensemble average. By imposing adequate selections of the investigated atoms, partial PDFs between individual species can be analyzed.

PDF data obtained for instance via MD simulations can be directly compared to their experimental counterpart, which are derived from synchrotron X-ray total scattering measurements performed on the CRISTAL beamline at the SOLEIL synchrotron facility using a monochromatic beam of wavelength λ = 0.51309 Å (energy *E* = 24.164 keV) and the second generation microstrip detector Mythen II. The PDFgetX2 program^59^ was used to correct and normalize the measured data and to obtain the experimental PDFs, *G*(*r*), by sine Fourier transform of the reduced reciprocal space total scattering function *F*(*Q*) = *Q*[*S*(*Q*) – 1] via:
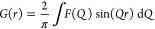
3In this study, the experimentally and theoretically determined Zn–Zn, Zn–O, and C–C PDFs of MOF-5 have been compared.

## 3 Results and Discussion

The accuracy of the employed SCC DFTB/3ob and GFN2-xTB methods depends strongly on the parametrization strategy of the individual contributions to the respective element pair interactions. Naturally, this has a dramatic influence on the structural description of the investigated systems and the quality of the resulting diffractograms. Overall, the structural description of the six investigated MOF systems did not suffer from the treatment at elevated temperatures, and none of the investigated systems displayed a tendency of the individual pores/channels collapsing. On the contrary, in almost all cases the application of the SCC DFTB approach results in a reduction of the lattice constants, which are then closer to the experimentally determined reference values compared to the energy-minimized geometries (i.e., 0 K conditions). The performance of the GFN2-xTB approach seemed to be slightly inferior to the SCC DFTB treatment in case of the Zn-based MOF systems. On the contrary, strong deviations from the SCC DFTB results and the experimental reference have been observed in case of MIL-68(Al) and MIL-53(Al) when treated at GFN2-xTB level.

This behavior is reflected in the individual PXRD patterns, with the intensity proportions of adjacent reflections being more accurate and peaks tend to shift toward the experimental 2θ values in case of the MD-averaged PXRD patterns. Albeit these shifts of the individual reflexes appear to be minor, they still reflect a notable improvement in the structural description with respect to the experimental reference. Additionally, several low intensity reflections became visible in the MD-averaged XRD patterns that are missing in the diffractograms of the 0 K structures. This can be explained by the influence of structural elements featuring rotatable or/and vibrating groups (e.g., the Zn_4_O^6+^ units in MOF-5) showing configurations displaced from the respective minimum structure in the MD simulations. In contrast, these structural entities are only represented via a single atomic configuration in the absence of thermal contributions.

### 3.1 Lattice Constants

#### 3.1.1 DFTB

In this section, the lattice constants obtained for different target systems via cell optimization as well as averaging over the MD trajectories are presented. The respective time evolution plots of the lattice parameters over the individual MD trajectories along with the respective powder X-ray diffractograms obtained at the different simulation settings *k*_*max*_ = 0, 1, and 2 are depicted in Figures S21–S30. In Tables 2 and 3 the lattice constants obtained from the different calculation methods and settings are summarized. As the MD simulation approach explicitly accounts for the impact of thermal and pressurization effects to resemble standard laboratory conditions (i.e., 298.15 K and 1.013 bar), the negative thermal expansion coefficients typical for a large number of MOF systems is well reflected by an associated increase of the respective unit cell. In particular, the systems MOF-5, DMOF-1, and MIL-68(Al) correctly display this characteristic when comparing the average lattice constants determined from the MD simulations to those obtained via energy minimization. In the case of the ZnZn-RPM system slightly larger cell parameters are observed over the simulation carried out using *xy*-isotropic pressure coupling (i.e., variations in the lattice parameters *a* and *b* remain coupled), whereas strongly deviating lattice constants are obtained when a semi-isotropic coupling (i.e., all three lattice parameters are varied independently) is employed. Note that original publication of this system provides no information about the sign of the associated thermal expansion coefficients.^60^ On the other hand, the ZIF-8 system features a positive thermal expansion in agreement with published data.^61^

**Table 2:** Unit Cell Parameters for MOF-5 and DMOF-1 Obtained via Energy Minimization (opt) and Averaging over the MD Trajectory (avg.) at SCC DFTB/3ob and GFN2-xTB Level in Comparison to the Experimental Reference Data

system	cell size	parameter set	*k*_max_	*a*/Å	*b*/Å	*c*/Å
MOF-5	1·1·1	avg. 3ob	2	26.143		
		avg. 3ob	1	26.130		
		avg. 3ob	0	26.134		
		avg. GFN2	1	25.287		
		opt. 3ob	2	26.270		
		opt. 3ob	1	26.270		
		opt. 3ob	0	26.270		
		opt. GFN2	1	25.398		
		exp. 258(2) K^62^		25.8320(5)		
						
DMOF-1	2·2·2	avg. 3ob	2	11.070		9.529
		avg. 3ob	1	11.076		9.532
		avg. 3ob	0	11.071		9.531
		avg. GFN2	1	10.752		9.487
		opt. 3ob	2	11.187		9.558
		opt. 3ob	1	11.173		9.544
		opt. 3ob	0	11.172		9.544
		opt. GFN2	1	10.828		9.486
		exp.^73^		10.93		9.61

**Table 3:** Unit Cell Parameters for ZIF-8 and ZnZn-RPM Obtained via Energy Minimization (opt) and Averaging over the MD Trajectory (avg.) at SCC DFTB and GFN2-xTB Level in Comparison to the Experimental Reference Data^a^

system	cell size	parameter set	*k*_max_	*a*/Å	*b*/Å	*c*/Å
ZIF-8	1·1·1	avg. 3ob	2	17.073		
		avg. 3ob	1	17.049		
		avg. 3ob	0	17.042		
		avg. GFN2	1	16.700		
		opt. 3ob	2	16.991		
		opt. 3ob	1	16.991		
		opt. 3ob	0	16.991		
		opt. GFN2	1	16.672		
		exp. 298 K^61^		17.0095(8)		
						
ZnZn-RPM	1·1·1	avg. semi 3ob	2	17.299	16.528	22.435
		avg. xy 3ob	2	16.966	16.836	22.433
		avg. xy 3ob	1	16.970	16.840	22.418
		avg. xy 3ob	0	16.970	16.840	22.420
		avg. xy GFN2	1	16.457	16.574	21.895
		opt. 3ob	2	16.909	16.927	22.462
		opt. 3ob	1	16.911	16.931	22.465
		opt. 3ob	0	16.911	16.931	22.465
		opt. GFN2	1	16.543	16.576	22.121
		exp. 100 K^60^		16.598(2)	16.643(2)	22.494(3)

a In case of ZnZn-RPM simulations semi- as well as *xy*-isotropic pressure coupling has been employed.

In the case of cubic MOF-5, the lattice constants of the optimized unit cell differ approximately 1.5% from the measured experimental lattice parameters, which have been determined as 25.8320 Å at 258.2 K.^62^ When invoking the MD treatment at elevated temperatures, the lattice constant decreases by approximately 0.5%, thus deviating only by about 1.0% from the experimental value. This behavior was found to be effectively independent of the employed setting for the *k*_*max*_-value, implying that a BZ integration considering only the Γ-point is perfectly adequate in this case. Considering that the employed 3ob DFTB parametrization was designed for the treatment of organic and bio-organic molecules (including a small number of metals to represent active sites in enzymatic systems), the near-perfect agreement between the calculated and experimentally determined lattice constants at elevated temperature seems remarkable. The latter can be explained by the fact that the functional groups of the BDC-linkers and the associated metal coordination in MOF-5 show high similarities to the binding motifs found in Zn-binding sites of enzymatic systems. In addition, the large pore volume of MOF-5 can to some extend be interpreted as gas-phase conditions rather than a condensed phase environment typical for dense solid-state systems.

The next target considered is DMOF-1, which already displays a much higher complexity. In addition, to its tetragonal crystal structure featuring two different lattice constants, it is also a binary MOF containing two different linking units. While the optimized MOF-5 unit cell does not change in size in dependence of the *k*_*max*_-setting, DMOF-1 displays minor changes in the lattice parameters with respect to the employed BZ integration (see Table 2). Again, the MD-averaged lattice constants remain closer to the experimental reference, deviating only by 1.3% (*a*) and 0.6% (*c*) in comparison to the optimized cell parameters differing by approximately 2% (*a*) and 0.008% (*c*), respectively.

The ZIF-8 system, a MOF of the zeolitic imidazolate framework type, features methylimidazolate units bound to Zn^2+^ atoms leading to a tetahedral coordination polyhedron forming the metal–organic framework. Compared to the experimental cubic lattice constant the parameter *a* increases as well in the performed simulations. The individual deviations from the experimental result are 0.1% (optimized) and 0.4% (averaged) suggesting no improvement was achieved in the description of the lattice constants.

The most complex Zn-based MOF containing two catalytically active porphyrin-groups is the ZnZn-RPM system. Comparison of the respective lattice parameters obtained via energy minimization and SCC DFTB MD simulations imply, that this MOF may too feature a positive thermal expansion coefficient. Noting that the experimental measurement was carried out at a low temperature of 100 K, it is reasonable that the optimized unit cell constants are closer to the measured values.^60^ In fact, the reported crystal structure of the orthorhombic ZnZn-RPM system displays two highly similar lattice constants along the *a* and *b* axes being 16.598(2) and 16.642(2) Å, the respective difference amounting only to 0.044 Å. When executing an SCC DFTB MD simulation employing semi-isotropic pressure coupling (i.e., the lattice constants *a*, *b*, and *c* are adjusted independently), a significant increase in the *a*-parameter to 17.299 Å was observed, corresponding to a 4% increase. This significant deviation is most likely the result of employing a simulation system comprising only a single unit cell. As a consequence, each rotation of the porphyrin-groups within the MOF structure can be expected to induce an artificial symmetry in the periodic QM treatment. For this reason a second simulation setup employing *xy*-isotropic coupling adjusting the *a*- and *b*-parameters in unison was investigated resulting in significantly improved lattice constants deviating only by about 1% from the experimental reference. Similar deviations were observed for the optimized ZnZn-RPM structures. However, the energy-minimized structures displayed a tendency to equalize the *a*- and *b*-parameters reducing the respective difference to approximately 0.02 Å. Similarly, as in the case of DMOF-1 very minor changes in the lattice parameters are observed for the different *k*_max_ settings.

Since aluminum is not considered in the 3ob parameter set,^63,64^ all DFTB calculations for MIL-68(Al) had to be carried out using the matsci parametrization (Table 4),^65,66^ which belongs to the family of second order DFTB methods. As a consequence of the comparably large size of the unit cell (total number of valence electrons of 2520) and the results obtained for the above-mentioned MOF systems, the *k*_*max*_ = 2 setting was not considered in this case. Comparison of the average lattice constants obtained via MD simulations against their energy minimized counterparts once more highlight the impact of a negative thermal expansion coefficient. Similarly as for the previous systems the deviation between the experimental lattice constant and both the optimized and average values remained within a margin of approximately 1%.

**Table 4:** Unit Cell Parameters for MIL-68(Al) and MIL-53(Al) Obtained via Energy Minimization (opt) and Averaging over the MD Trajectory (avg.) at SCC DFTB and GFN2-xTB Level in Comparison to the Experimental Reference Data

system	cell size	parameter set	*k*_max_	*a*/Å	*b*/Å	*c*/Å
MIL-68(Al)	1·1·3	avg. matsci	1	21.200	35.988	6.769
		avg. matsci	0	21.195	35.979	6.769
		avg. GFN2	1	20.251	33.201	6.606
		opt. matsci	1	21.227	36.066	6.792
		opt. matsci	0	21.217	36.082	6.806
		opt. GFN2	1	21.037	34.971	6.638
		exp.^74^		20.51(1)	35.92(1)	6.683(1)
						
MIL-53(Al)	2·1·1	avg. matsci	1	6.767	16.787	12.992
		avg. matsci	0	6.766	16.803	12.970
		avg. GFN2	1	6.458	17.222	11.319
		opt. matsci	1	6.789	16.853	12.945
		opt. matsci	0	6.804	16.500	13.408
		opt. GFN2	1	6.730	17.710	10.964
		exp. 548 K^68^		6.608(1)	16.675(3)	12.813(2)

As for the previously mentioned system, the topologically similar MIL-53(Al) system, all calculations have been carried out using the matsci^65,66^ parameter set (Table 4). In contrast to MIL-68(Al) this metal–organic framework features a significantly lower number of atoms in the unit cell (684 vs 152). The experimental lattice constants *a*, *b*, and *c* deviate approximately 1.5% from either the respective averaged and optimized ones. Considering the very low value of the thermal expansion coefficient according to published data^67^ and the temperature of 548 K present at the experimental PXRD measurement of the reference data^68^ the results appear highly adequate.

#### 3.1.2 GFN2-xTB

As discussed above the parametrization of the GFN2-xTB method is based on reference data for a large set of nonperiodic molecular systems *in vacuo*. In order to assess whether the internal vacuum environment associated with the large pores and channels of MOF systems permits a transfer of the parametrization to the class of MOF systems, energy minimizations as well as MD simulations have been carried out. Due to the increased computational demand of the GFN2-xTB method and based on the findings of the SCC DFTB/3ob calculations for the MOF-5, DMOF-1, ZIF-8, and ZnZn-RPM systems presented above, a BZ sampling employing *k*_max_ = 2 was not considered in this case.

Overall, calculations utilizing GFN2-xTB result in smaller lattice constants than those observed in the SCC DFTB calculations irrespective of the employed parameter set. More specifically, in case of the MOF-5 the lattice constant differs by approximately 3.3% (MD average) and 3.4% (optimized structure) from the DFTB results and by 2.1% (average) and 1.7% (optimized) from the experimental reference value (Monkhorst–Pack sampling using *k*_max_ = 1).

The theoretical treatment of the DMOF-1 system via GFN2-xTB also leads to higher deviations in the averaged lattice constants. While the unit cell parameters *a* and *c* of the optimized structure deviate only by about 1.1% from the experimental value, the deviation in the average values of *a* and *c* is increased to 1.4%.

Similarly, as the above-mentioned systems the lattice constant of ZIF-8 also deviates about 1.9% (averaged) and 2.0% from the experimental value indicating only a small improvement compared to the optimization.

Since only *xy*-isotropic pressure coupling resulted in an adequate description of the ZnZn-RPM system, only this setting was considered for simulations employing the GFN2-xTB approach. The resulting values for the lattice constants differ approximately by 1.0% from the experimental reference, thus achieving a similar overall accuracy as observed in the SCC DFTB treatment. However, in contrast to the DFTB-based values, which increased in the *in silico* modeling, the lattice constants are consistently lower in the GFN2-xTB case when compared to the experimental results. Nevertheless, the use of only a single unit cell and a *k*_*max*_ = 1 setting in combination with GFN2-xTB still provides a reasonable description of the experimental values.

Similarly, as observed for the Zn-based systems the optimized unit cell of MIL-68(Al) features smaller lattice constants when employing the GFN2-xTB method in comparison to the SCC DFTB/3ob approach. While the optimized unit cell deviates by about 2.5% from the experimental values, the MD-averaged lattice constants shows a larger deviation of 3.5%. These comparably large deviations can be attributed to a nonideal parametrization of aluminum compared to the *matsci* parameter set, since the former has been designed with focus on the treatment of purely organic and biomolecular systems.

Unsurprisingly, the second investigated Al-based system MIL-53(Al) again features the tendency to smaller lattice constants when employing the GFN2-xTB method. The deviations from the experimental value are even larger with values of 4.1% (average) and 3.1% (optimized). As mentioned above, the parameters are probably less ideal for investigation of systems containing Al-species.

### 3.2 X-ray Diffraction Patterns

A particularly useful analysis enabling a more detailed comparison of the structural properties to experimental reference data is the calculation of the associated X-ray diffractograms. In addition to the typically employed minimum structure the averaged XRD patterns evaluated over regular intervals of the MD simulation trajectories have been considered. Based on the very good agreement of the average lattice constants obtained via molecular dynamics and the experimentally determined values (especially when employing the SCC DFTB approach), a similar increase in accuracy can be expected for the associated XRD data.

#### 3.2.1 MOF-5

**Figure 3:**
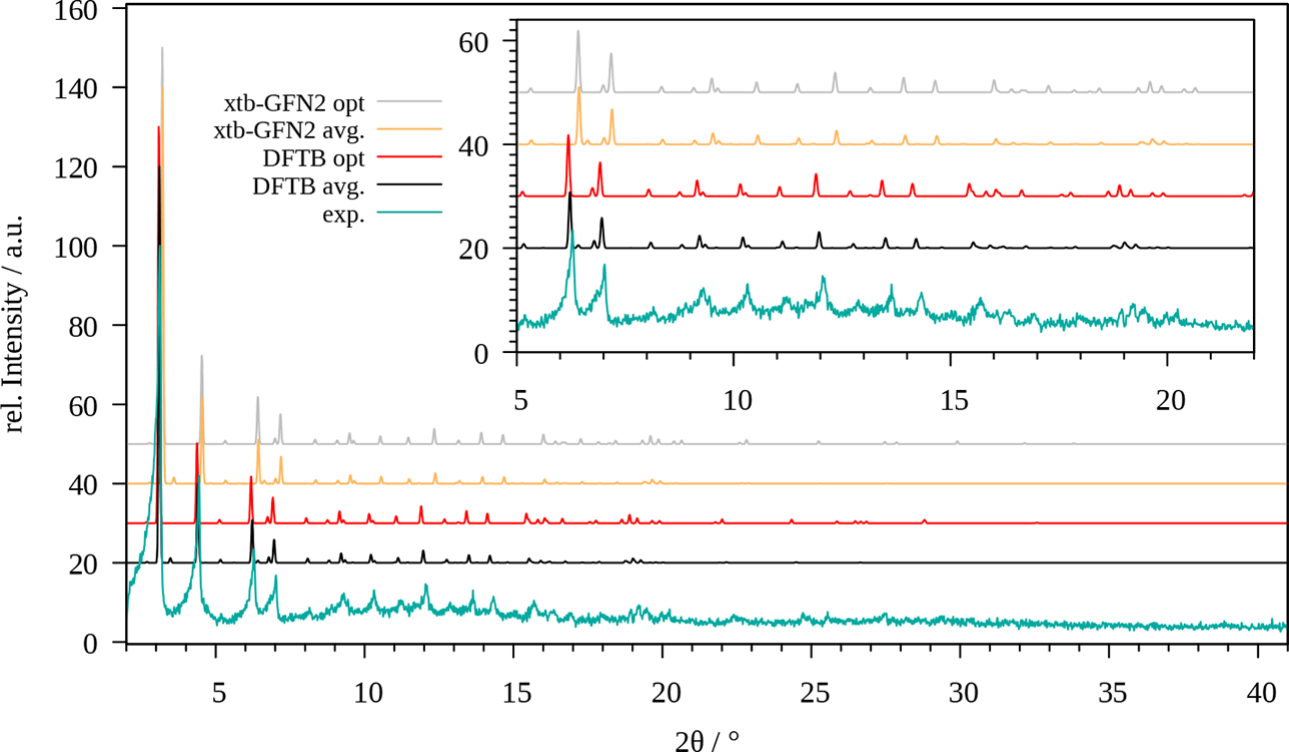
Comparison of the experimental powder X-ray diffractogram of MOF-5 (cyan) with those obtained for the optimized system (red, gray) and via averaging over the MD trajectory (black, yellow) obtained at SCC DFTB/3ob and GFN2-xTB level, respectively.

Figure 3 shows a comparison of the XRD patterns obtained for the minimized structure as well as via averaging over the MD trajectory at SCC DFTB/3ob and GFN2-xTB level against the experimental reference. While overall no major changes in the main signals seem to be present, close investigation of the reflexes in the range from 5 to 20° reveals a number of changes. For both investigated calculation methods the averaged XRD patterns are in very good agreement with that observed from the minimum configuration. This confirms that aside from the small changes in the lattice parameters the structural properties are maintained even though the system is treated at elevated temperatures in the MD simulations.

Irrespective of the applied level of theory, the individual peaks show an increased thermal broadening along with a reduction in their intensity in the MD treatment as expected. However, a small number of reflexes not present in the XRD patterns of the optimized structure are visible in their MD counterpart (e.g., the small signals visible at 6.4° (SCC DFTB/3ob) and 6.6° (GFN2-xTB), respectively). This implies that certain reflexes are not accessible when employing high-symmetry minimum structures, which represents a further benefit achieved in the more demanding MD treatment.

Overall, it can be seen that the SCC DFTB/3ob level provides a highly accurate representation of MOF-5, while the individual reflexes are consistently shifted to larger angles in both the optimized and averaged pattern when employing the GFN2-xTB approach, which corresponds well to the observation of an increase in the lattice parameters already observed in the cell optimization.

#### 3.2.2 DMOF-1

**Figure 4:**
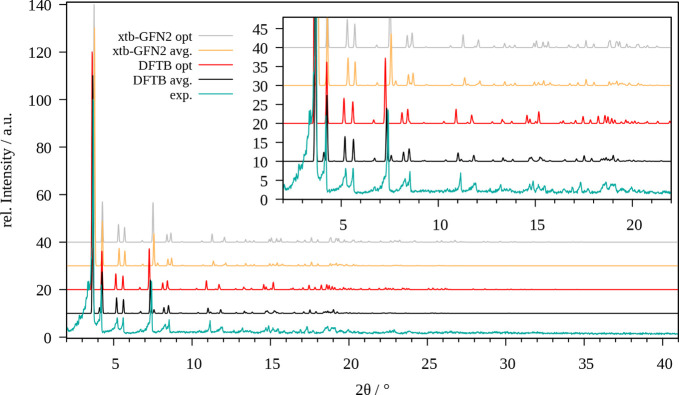
Comparison of the experimental powder X-ray diffractogram of DMOF-1 (cyan) with those obtained for the optimized system (red, gray) and via averaging over the MD trajectory (black, yellow) obtained at SCC DFTB/3ob and GFN2-xTB level, respectively.

The comparison between the experimental and theoretically determined XRD patterns for DMOF-1 is shown in Figure 4. Similarly as observed for MOF-5 the negative thermal expansion coefficient of the DMOF-1 system is correctly represented by small shifts of the reflexes to larger angles in the MD averaged patterns in comparison to the optimized case. As before, the experimental intensities and reflection angles are more adequately resembled by the MD averaged pattern determined via SCC DFTB implying that the 3ob parameter set is very well-parametrized. While individual reflections are not ideally represented (e.g., the low intensity reflex at 7.5°, which is missing in the both experimental as well as the optimized pattern), several features are notably improved, such as the reflections in the range of 15 and 20°. Again, the GFN2-xTB-based patterns are consistently shifted to higher 2θ values, which is in agreement with the smaller unit cell obtained in the optimization and the MD simulations. Nevertheless, the overall shape of the experimental pattern is retained, although individual features are either missing or resembled in a less-accurate way (e.g., the low intensity reflections in the range of 18–19°). Curiously, the signals in this area are present in the patterns of both the SCC DFTB and GFN2-xTB optimized structures.

#### 3.2.3 ZIF-8

**Figure 5:**
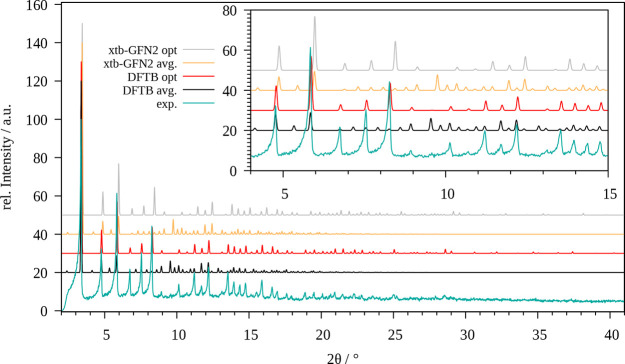
Comparison of the experimental powder X-ray diffractogram of ZIF-8 (cyan) with those obtained for the optimized system (red, gray) and via averaging over the MD trajectory (black, yellow) obtained at SCC DFTB/3ob and GFN2-xTB level, respectively.

The calculated and experimentally measured PXRD patterns of the ZIF-8 system are depicted in Figure 5. In comparison to the results obtained for the of optimized structures the respective averaged patterns feature more individual peaks. The averaged patterns also shift slightly to smaller 2θ values due to the larger unit cell parameters, although the overall unit cell size remains conserved during the simulation in comparison to the optimized structure. The associated values are listed in Table 3. Overall, the performance of the SCC DFTB/3ob level seems superior compared to GFN2-xTB as the respective shape differs less from the experimental reference.

#### 3.2.4 ZnZn-RPM

**Figure 6:**
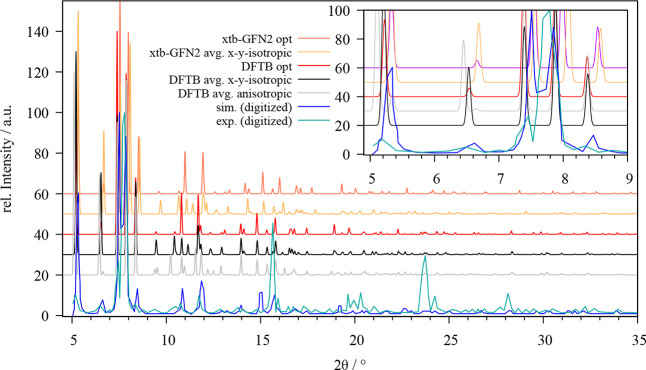
Comparison of the experimental (cyan) and simulated (blue) powder X-ray diffractograms of ZnZn-RPM provided by Farha et al.^60^ with those obtained for the optimized system (red, yellow) and via averaging over the MD trajectory in case of semi-isotopic (gray) and *xy*-isotropic (black, orange) pressure coupling obtained at SCC DFTB/3ob and GFN2-xTB level, respectively.

As already discussed above the ZnZn-RPM lattice constants *a* and *b* show notable divergence from the experimental values in case a semi-isotropic pressure coupling is employed in the MD simulation. In order to prevent the observed deformation of the unit cell, the coupling of the *a*- and *b*-parameters was carried out in unison (i.e., *xy*-isotropic coupling), which resulted in a more adequate description of the unit cell over the simulation time. Figure 6 depicts the associated X-ray diffractograms obtained via optimization as well as via MD simulations at both SCC DFTB/3ob and GFN2-xTB level in comparison to the reported experimental and simulated XRD patterns.^60^

While the optimized and *xy*-isotropic patterns feature similar reflections the diffractogram obtained via semi-isotropic pressure coupling tend to split a number of reflections into two or more smaller signals. In the range of 8–10.5° two reflexes are present in the experimental pattern, which are adequately reproduced in their MD-averaged counterparts, whereas these reflexes seem to be absent (or at least shifted to different values while featuring significantly reduces intensities) when employing the energy-minimized structures. The intense reflections at approximately 15.5 and 23.5° appear to be measurement artifacts as the respective intensities in all other patterns (including the simulated XRD pattern reported in the same work)^60^ are negligible in comparison.

#### 3.2.5 MIL-68(Al)

**Figure 7:**
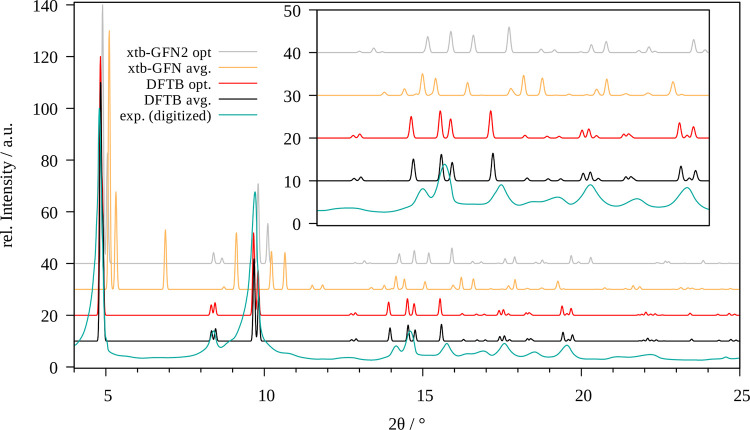
Comparison of the experimental powder X-ray diffractogram of MIL-68(Al) reported by ref (56) (cyan) with those obtained for the optimized system (red, gray) and via averaging over the MD trajectory (black, yellow) obtained at SCC DFTB/3ob and GFN2-xTB level, respectively.

Figure 7 depicts the XRD patterns obtained for the optimized geometry and via MD averaging at SCC DFTB/matsci level are compared to the experimental reference reported by Embrechts and co-workers^56^ Due to the strong complexation of the polarizing aluminum ions by the oxygen atoms of the BDC^2–^ linker units, the framework features only modest flexibility at room temperature. As seen earlier, the treatment at elevated thermal conditions has only a very minor influence on the size of the unit cell in this case, which is also reflected by nearly identical patterns in both the optimized and MD-averaged diffractograms. In both cases, the characteristics of the experimental XRD pattern are well reproduced, although the latter features a comparably large broadening of the peaks representing the individual reflexes.

However, in this case the X-ray diffraction patterns obtained from the GFN2-xTB treatment lead to strongly differing results compared to the experimental reference. As mentioned already above the unit cell parameters deviate significantly from the experimental and SCC DFTB values, which in turn leads to notably shifted reflexes in the XRD patterns in the optimized and averaged case. Comparing the overall shape of the patterns to the experimentally data, the optimized pattern resembles the latter quite well for most of the reflexes, while the MD-averaged diffractogram features reflexes with no matching signal in the experimental reference (e.g., 2θ angles near 7, 9, and 11°).

#### 3.2.6 MIL-53(Al)

**Figure 8:**
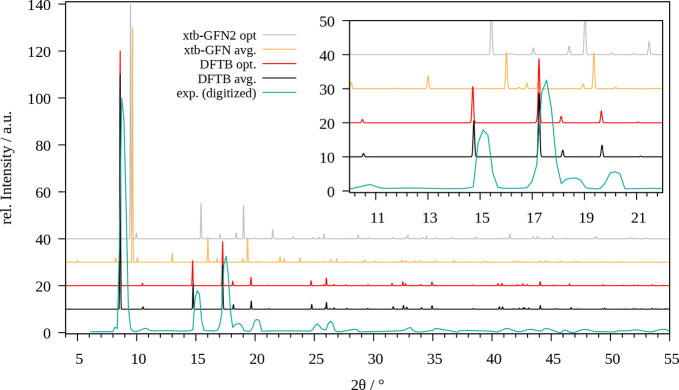
Comparison of the experimental powder X-ray diffractogram of MIL-53(Al) reported by ref (68) (cyan) with those obtained for the optimized system (red, gray) and via averaging over the MD trajectory (black, yellow) obtained at SCC DFTB/3ob and GFN2-xTB level, respectively.

In Figure 8 the corresponding XRD patterns obtained for the minimum structure and via MD averaging are depicted. In case of the SCC DFTB/matsci treatment, both the averaged and optimized pattern, show only slight deviations probably due to the very small value of the thermal expansion coefficient. In contrast, the patterns calculated of structures resulting from the GFN2-xTB treatment show large deviations when compared to the experimental pattern. However, the averaged PXRD pattern of the configurations extracted from the GFN2-xTB MD simulation deviates less from the experimentally measured data.

### 3.3 Pair Distribution Functions

A particularly useful analysis enabling a direct comparison between experimental measurements and theoretical simulation data are atomic pair distribution functions. In this work, PDFs for the MOF-5 systems obtained via measurements performed on the CRISTAL beamline at the SOLEIL synchrotron facility are compared to their theoretical counterparts obtained via MD simulations at SCC DFTB/3ob and GFN2-xTB level, respectively.

**Figure 9:**
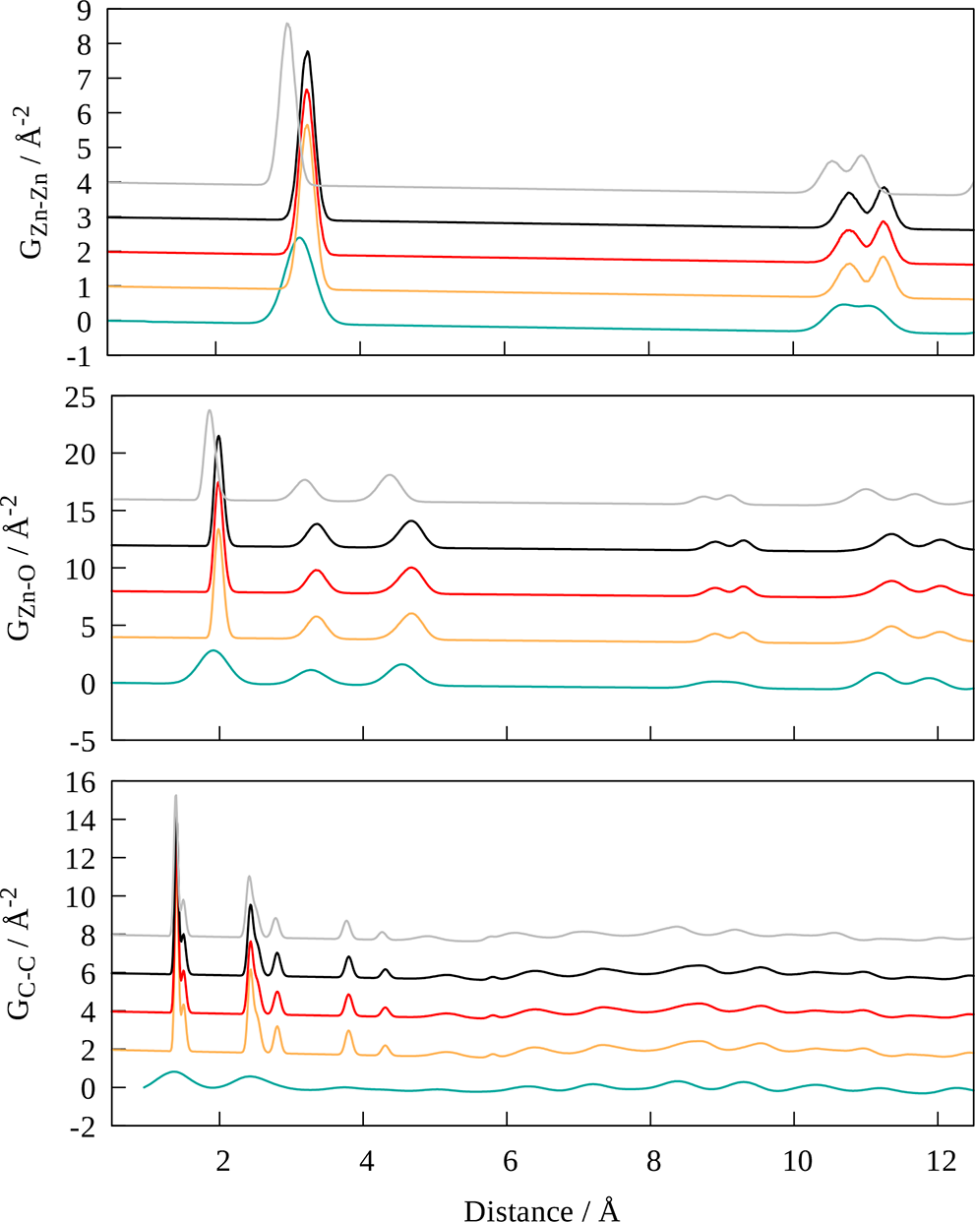
Comparison of atom of the respective element pairs Zn–Zn (top), Zn–O (center), and C–C (bottom) obtained via SCC DFTB/3ob MD simulations of MOF-5 using *k*_max_ = 2 (black), 1 (red), and 0 (Γ-point sampling, yellow) against the experimental reference (blue). The corresponding PDFs obtained at GFN2-xTB level (*k*_max_ = 1) are shown as well (gray).

In Figure 9 the respective Zn–Zn, Zn–O, and C–C PDFs obtained for MOF-5 are depicted. At first sight, a notable difference in peak intensity can be identified, which can be linked to the dramatically different system sizes in the experimental and theoretical investigations. While in the simulations only a single unit cell was considered, the experimental measurements are carried out employing a macroscopic system in the (sub)molar range.

When considering the PDFs obtained at SCC DFTB/3ob level, effectively identical distribution functions are obtained irrespective of the employed *k*_max_ setting representing the accuracy of the BZ integration. Compared to the experimental PDFs, the first peaks of the Zn–Zn as well as Zn–O distribution are shifted to slightly larger values by approximately 0.05–0.1 Å. On the other hand, peaks at larger distances near approximately 11 Å representing neighboring Zn_4_O clusters show a better agreement in line with the excellent representation of the unit cell parameters discussed above. Although the peak broadening and associated low-intensities observed in the experimental C–C PDFs makes their comparison more challenging, all main features are well captured in the theoretical distributions as well.

Since a minimal setting for the BZ treatment proved sufficient, only the *k*_max_ = 1 case was considered for the GFN2-xTB MD simulation. It can be seen in Figure 9 that the peaks in both the Zn–Zn as well as the Zn–O distribution are consistently shifted to smaller radii, indicating shorter average Zn–O distances correlating with the reduced size of the unit cell discussed above. On the other hand, the C–C PDFs show nearly identical features to the SCC DFTB/3ob case, especially in the range from 1 to 5 Å.

This comparison provides direct evidence that both considered DFTB methods provide an adequate description of the MOF-5 system at elevated temperatures, since the PDFs provide detailed insight into the distributions of individual atom pairs at various distances in the system.

### 3.4 Structural Collapse in MIL-68(Ga)

For all examples considered so far the SCC DFTB/3ob/D3 level resulted in a highly accurate structural description of the target systems at elevated temperature being visible both in terms of the respective lattice parameters as well as the excellent agreement of the MD-averaged XRD patterns with the corresponding experimental reference. The latter provide a direct indication that the relative distances of the atoms within the simulation systems are correctly distributed, which cannot be concluded if just the respective lattice constants are considered.

**Figure 10:**
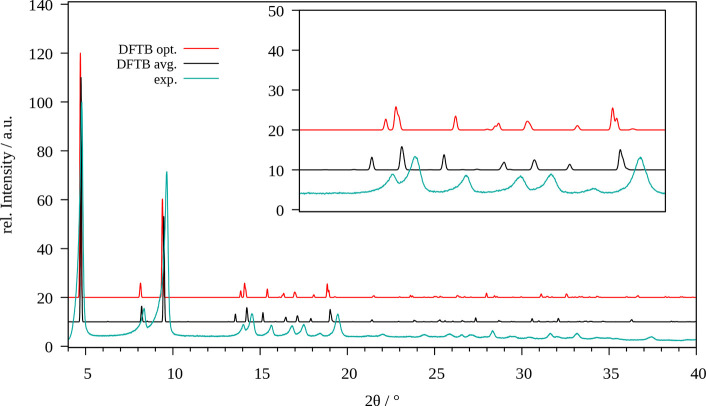
Comparison of the experimental powder X-ray diffractogram of MIL-68(Ga) (cyan) with those obtained for the optimized system (red) and via averaging over the MD trajectory (black) obtained at SCC DFTB/3ob and GFN2-xTB level, respectively.

An example for a nonideal case is the MIL-68(Ga) system, sharing the same crystal structures as its Al-containing counterpart discussed above. While DFTB parameters for Ga are available within the mio set,^69^ the main focus in this case was laid on the treatment of GaAs surfaces.^70,71^ It is therefore not clear whether this Ga parametrization provides a suitable choice for the treatment of the formally three times positively charged Ga^3+^ ion (Figure 10). Execution of an energy minimization of the MIL-68(Ga) considering both the atomic positions as well as the lattice parameters results in an adequate description of the system, thereby retaining all features of the nanoporous structure. The respective lattice parameters amount to *a* = 21.763 Å, *b* = 37.461 Å, and *c* = 6.775 Å and are slightly enlarged compared to the experimental values reported by Volkringer et al. of *a* = 21.176 Å, *b* = 36.703 Å, and *c* = 6.7423 Å.^72^ Despite these deviations in the range of 0.5–2.8% the results imply that the combined mio + Ga parametrization can be in principle applied for the treatment of the MOF systems (i.e., despite the focus on semiconducting compounds the parameter set is capable of describing ionic Ga-species).

However, when executing a molecular dynamics simulation at standard conditions, a continuous drift in the lattice constant from the experimentally determined values is observed (see Figure S31). This unphysical phase transition can be interpreted as an amorphization of the MOF induced by the collapse of the nanoporous structure and consequently, this simulation setup cannot be employed to study the properties of MIL-68(Ga) and its interaction with guest molecules. This example provides clear evidence that although a correct minimum structure can be identified the associated parametrization is not necessarily capable of providing a correct description at nonzero thermal conditions.

A potential alternative to a treatment under isothermal–isobaric conditions are simulations in the canonical ensemble, which can be achieved by imposing a constant volume (i.e., fixed lattice constants). A particular obstacle of such a simulation strategy rests with the choice of suitable lattice parameters, which have to be provided as input. Due to the structural similarity the relative difference in the lattice constants of MIL-68(Al) has been employed for orientation. In this case the cell parameters *a*, *b*, and *c* decreased by 0.12, 0.25, and 0.44% when comparing the optimized and MD-averaged data. Assuming similar ratios in the lattice constants of the topologically similar MIL-68(Ga) system, reasonable estimates amounting to *a* = 21.5856 Å, *b* = 37.2274 Å, and *c* = 6.9598 Å are obtained.

Execution of the simulation at 298.15K and constant volume conditions results in a stable trajectory with no indication of a structural collapse. The comparison of the XRD patterns obtained for the minimum geometry and via MD-averaging depicted in Figure 10 shows an overall very good agreement with the experimental reference. For larger 2θ values, a notable shift in the reflexes is observed, albeit the relative positioning and the intensity of the pattern still aligns with those observed in the measurement. Thus, although the lattice constants employed in the simulation show some minor deviations, the internal structural motifs of MIL-68(Ga) are retained. This finding implies that a constant volume simulation setup can be employed as a fallback strategy when aiming at investigations of guest molecules embedded in this particular MOF material. However, more subtle features such as the breathing effect prominent in a large number of nanoporous functional materials cannot be represented. In this case, an adjustment of the DFTB parameters is required to adequately describe the system subject to an external pressure.

## 4 Conclusion

The data presented in this study provides manifold insight into the applicability of DFTB-based methods for the description of metal–organic frameworks. Although several studies have provided clear evidence that the SCC DFTB/3ob and matsci parametrizations can indeed provide equilibrium geometries in good agreement with experimental reference data, these findings provide no information with respect to the description of these systems at elevated temperatures. However, due to the highly advantageous cost-accuracy ratio of DFTB methods in combination with an increased MD time step featured by the application of a rigid-body treatment of hydrogen-containing bonds, sufficiently long simulation trajectories can be achieved to assess the performance of DFTB methods for the treatment of MOF structures at nonzero thermal conditions and external pressures.

The simulation studies carried out at standard conditions clearly demonstrate that characteristic properties such as the negative thermal expansion coefficients identified for a large number of MOFs are equally well represented as the experimental X-ray diffraction patterns and pair distribution functions determined via synchrotron measurements. While PDFs can be directly calculated from the simulation trajectory, X-ray diffraction patterns at elevated temperatures can be obtained via averaging of several (in this study 5000) individual XRD patterns calculated for individual structural snapshots extracted from the trajectory at regular intervals. Both the PDF as well as the XRD analysis provide manifold insight with respect to the atomic distribution inside the investigated systems, that cannot be accessed via a simple comparison of the respective lattice parameters.

In particular, the SCC DFTB/3ob and matsci levels applied to the different MOF systems provide a highly adequate description of the target systems despite the efficiency of this approach being at least 2 orders of magnitude faster when compared to a corresponding DFT treatment. Similarly, the GFN2-xTB level initially parametrized with focus on nonperiodic, (bio)organic systems was found to be remarkably robust in the investigation of the different Zn-based MOF systems. However, the deviations in the lattice parameters, XRD patterns and PDFs proved slightly larger compared to the employed SCC DFTB methods. However, the comparably good performance observed in the treatment of the Zn-based MOF systems could not be reproduced in case of MIL-68(Al) and MIL-53(Al). This demonstrates that the performance of the GFN2-xTB method is strongly dependent on the target system.

The MD simulation results of MIL-68(Ga) have demonstrated that the DFTB MD simulation approach is sensitive to the nature of the underlying parameter set. Considering that the main focus in the parametrization of Ga given to on the description of semiconducting compounds such as GaAs, it is to some extent surprising that the overall structural properties of MIL-68(Ga), formally containing trivalent Ga-ions in the SBU, can be adequately described in structure optimizations as well as constant volume MD simulations. However, in this case an adjustment of the DFTB parameters is required when aiming at the application of a simulation protocol facilitating external pressure (e.g., when studying more intricate properties as for example the bulk moduli and thermal expansion).

In the case of the latter properties, a number of independent MD simulations at different temperatures and pressures are required. While the outlined rigid-body DFTB MD simulation enables the execution of comparably long simulation times when compared to DFT-based approaches, it was demonstrated that these properties can be investigated with high accuracy when employing (polarizable) force fields. The SCC DFTB MD methodology on the other hand is highly suitable when introducing changes in the MOF systems. While in classical simulations typically an extensive parametrization of new residues is required, the inclusion of modifications to the linker units (e.g., by adding substituents such as aliphatic side chains,. halogens, or other functional groups such as −OH, −NH_2_, or −NO_2_) can be implemented in a straightforward manner within a DFTB MD framework with only little modifications of the associated input. Similarly, the inclusion of different guest molecules and mixtures thereof does not require any lengthy parametrization procedure.

In summary, this study provides clear evidence that the applied DFTB methodology provides a highly adequate alternative to represent MOF structures in MD simulations, which greatly extends the range for computational studies focused on guest@MOF systems. Considering the importance of the latter in a broad variety of disciplines which (in addition to the traditional storage of gaseous guest molecules in MOF structures) receives increasing interest in a variety of disciplines focused inter alia on photoactive functional materials, advanced battery research, and drug delivery, the findings presented in this study provide a key premise for the study of this highly versatile class of nanomaterials.

## Supporting Information Available

Details of the applied molecular dynamics approach including the respective simulation protocol; synthesis of MOF-5 and DMOF-1; Details of the experimental and theoretical determination of X-ray diffraction patterns (PDF)
