# Supplementary material for: Structural Properties of Metal–Organic Frameworks at Elevated Thermal Conditions via a Combined Density Functional Tight Binding Molecular Dynamics (DFTB MD) Approach
Source: J Phys Chem C Nanomater Interfaces. 2023 Jan 10;127(3):1560–75. doi: 10.1021/acs.jpcc.2c05103 (PMC9884096; doi:10.1021/acs.jpcc.2c05103)
Supplement: Supplementary file 1 — jp2c05103_si_001.pdf [file jp2c05103_si_001.pdf]

Structural Properties of Metal-Organic Frameworks at Elevated Thermal Conditions *via* a Combined Density Functional Tight Binding Molecular Dynamics (DFTB MD) Approach

Supplementary Material

Felix R. S. Purtscher[a], Leo Christanell[a], Moritz Schulte [a], Stefan Seiwald[a], Markus Rödl[a], Isabell Ober[a], Leah K. Maruschka[a], Hassan Khoder[b], Heidi A. Schwartz[a], El-Eulmi Bendeif[b], and Thomas S. Hofer[a*]

[a]Institute of General, Inorganic and Theoretical Chemistry
Center for Chemistry and Biomedicine
University of Innsbruck, Innrain 80-82, A-6020 Innsbruck, Austria
Tel.: +43-512-507-57111
Fax: +43-512-507-57199

[b] CRM2 UMR CNRS 7036
Université de Lorraine
F-54000 Vandæuvre-lès-Nancy, France
Tel.: +33-3-72-74-56-34
Fax: +33-3-72-74-52-18

January 10, 2023

*Corresponding author: t.hofer@uibk.ac.at

S1 Molecular Dynamics Simulations

All SCC DFTB MD simulations were carried out in the isothermal-isobaric (NPT) ensemble using the in-house developed molecular dynamics simulation software [1–4] interfaced to the DFTB+ program. [5–9] The SCC DFTB calculations of energy, forces and the associated stress tensors were performed under periodic boundary conditions employing the 3ob [10,10,11] and matsci [12,13] parameterization in case of the Zn- and Al-based MOFs, respectively. As required by the 3ob parameter set, a damping of all interactions between non-hydrogen and hydrogen atoms was applied, with the respective damping coefficient ζ_{XH} being set to 4.0. [10] In addition, to improve the description of dispersive interactions the D3 correction scheme [10] was applied in case of both the 3ob and matsci paramterizations. In case of all Zn-based MOFs considered in this work, Monkhorst-Pack sampling [14] was performed using a 2·2·2 ($k_{\mathrm{max}} = 2$) as well as a 1·1·1 ($k_{\mathrm{max}} = 1$) extension. Due to the large size of the unit cell in the MIL-68 case and for MIL-53(Al), only the latter setting was considered. In addition, all systems were also studied employing only Γ-point sampling (*i.e.* $k_{\mathrm{max}} = 0$).

In addition to the calculations employing the SCC DFTB method, all systems were also studied employing the recently released GFN2-xTB [15,16] implementation in the DFTB+ program package. The latter enables the application of this method in an periodic calculation environment. Since the parametrization of the GFNn-xTB methods was based on reference data for non-periodic molecular systems in vacuum, any application to periodic problems should be critically assessed. While metal-organic frameworks comprise periodic condensed matter systems, the associated large sizes of the pores and channels inherent to MOF structures can be interpreted as an internal vacuum environment. It is, thus, of particular interest to investigate, whether the good performance of the GFN2-xTB method for isolated molecular compounds can be transferred to the class of MOF systems. Due to the increased computational demand of the GFN2-xTB Hamiltonian, only Monkhorst-Pack sampling employing a 1·1·1 ($k_{\mathrm{max}} = 1$) grid as well as Γ-point sampling were considered in this work.

The velocity-Verlet algorithm [17,18] was employed to integrate the equations of motions. To facilitate an extended MD time step of 2.0 fs, holonomic constraints were applied to all bonds involving hydrogen atoms via the SHAKE/RATTLE algorithms. [19,20] The respective constraint distances have been determined based on preliminary MD simulations at the target conditions employing a shorter MD time step of 0.5 fs, thereby enabling full flexibility for all bonds in the systems. The coupling to an external heat reservoir was realized using the Nosé-Hoover chain thermostat with a chain length of 5, while constant pressure conditions were implemented based on the Berendsen manostat algorithm. [21]

The cell parameters $\{a, b, c\}$ for the orthorhombic systems MIL-68(Al) and MIL-53(Al) were allowed to vary independently over the simulation time (*i.e.* semi-isotropic pressure coupling), while the lattice constant $\{a\}$ of the cubic MOF-5 and ZIF-8 systems and the constants $\{a, b\}$ of the tetragonal DMOF-1 system were adjusted in unison (*i.e.* isotropic and xy-isotropic coupling). Note that for the ZnZn-RPM system an orthorhombic unit cell with highly similar values for the lattice vectors a and b amounting to 16.598 and 16.643Å was reported. [22] For this

reason two independent SCC DFTB MD simulations were carried out to assess the influence of pressure coupling along the a and b directions. In the first case all three lattice parameters a, b and c were adjusted independently (semi-isotropic), while in the second case the pressure coupling along the a and b axes was carried out in unison (xy-isotropic). The associated lattice angles $\{\alpha, \beta, \gamma\}$ remained fixed at 90° for all systems, over the course of the simulations.

Based on the size of the respective lattice constants in the unit cell, supercells of the individual systems were generated to ensure suitable system sizes, *e.g.* when aiming at the incorporation of guest molecules within the MOF structure.[23] For DMOF-1 a 2·2·2 supercell was employed, while in case of MIL-68(Al) an (1·1·3) configuration was used. Due to the larger size of MOF-5, ZnZn-RPM and ZIF-8 a single unit cell (*i.e.* 1·1·1) already proofed sufficient in these cases. The MIL-53(Al) system was enlarged to a (2·1·1) supercell and the modification with large pores (which can be stabilised for room temperature[24]) was chosen due to its orthorombic structure. After an initial heating to the target temperature, all systems have been subject to at least 5 000 MD steps (10 ps) of equilibration under NPT conditions, followed by sampling for at least 50 000 MD steps (100 ps).

S2 Experimental Part

S2.1 Synthesis of MOF-5 and DMOF-1

MOF-5 was synthesized following the protocol given in the literature.[25] 1.266 g terephthalic acid (7.62 mmol) and triethylamine (2.13 ml) were dissolved in 100 ml of DMF. 4.25 g $Zn(OAc)_2{\cdot}2H_2O$ (19.35 mmol) was dissolved in 125 ml DMF. While stirring, the zinc salt solution was added dropwise to the organic solution over 15 min. The mixture was stirred for 2.5 h. The precipitate was filtered off and immersed in 62.5 ml DMF overnight. It was then filtered off again and immersed in 87.5 ml $CHCl_3$. The solvent was exchanged three times over seven days. Finally, the precipitate was decanted. To remove the solvent completely, the resulting powder was heated at 100°C for 12 h under reduced pressure and stored under an argon atmosphere in the glovebox to prevent absorption of humidity and decomposition by contact with moisture.

Similarly, DMOF-1 was snythesized according to the protocol provided in the literature.[26] $Zn(NO_3)_2 \cdot 6\, H_2O$ (125.0 mg, 0.42 mmol), terephthalic acid (70.0 mg, 0.42 mmol) and dabco (1,4-diazabicyclo[2.2.2]octane) (20.0 mg, 21.0 mmol) were mixed with DMF (dimethylformamide) (3 ml) in an 8 ml Teflon lined autoclave. The mixture was heated (120°C, 2 days) in an oven and cooled down to room temperature afterwards. The resulting colourless powder was filtered, then washed with a small amount of DMF and dried on air overnight. To remove embedded DMF molecules, the residue was heated under reduced pressure (120°C , 24 h) and stored under argon atmosphere. The phase purity of DMOF-1 was checked by XRPD.

S2.2 Experimental determination of X-ray diffraction data

While the patterns of ZnZn-RPM and MIL-68(Al) were extracted from the respective literature,[22,27] the diffractograms of DMOF-1 and MOF-5 have been newly measured.

PXRD measurements were carried out on a Stoe Stadi P diffractometer (Stoe, Darmstadt, Germany) in transmission geometry with Mo-$K_{\alpha 1}$-radiation ($\lambda = 70.93$ pm) utilizing a focusing Ge(111) primary beam monochromator and a Mythen 2 DCS4 detector. The measurement was performed in the 2θ range of $2.0-40.4°$ with a step size of $0.015°$. The respective powder was sealed in a glass capillary under argon atmosphere to prevent absorption of humidity. The respective X-ray wave lengths employed in the different studies are summarised in Tab. ??.

S3 MOF-5

S3.1 Time evolution of lattice parameters

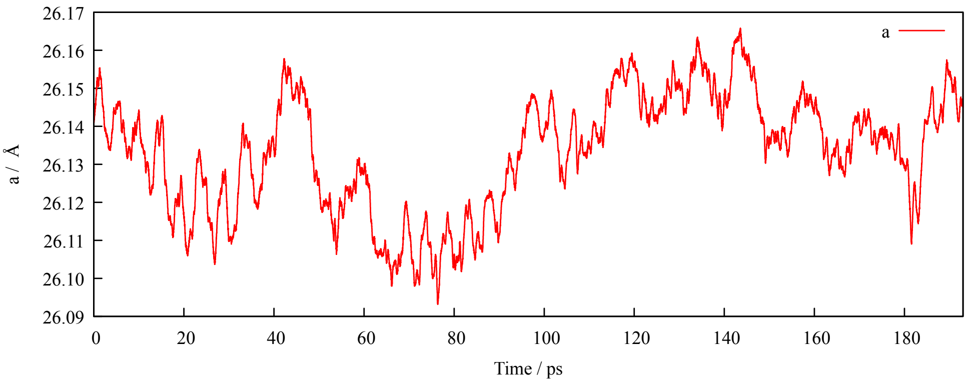

Figure S1: MOF5 cubic lattice constant a changes during the MD Simulation with respect to the k-point sampling (k=2)

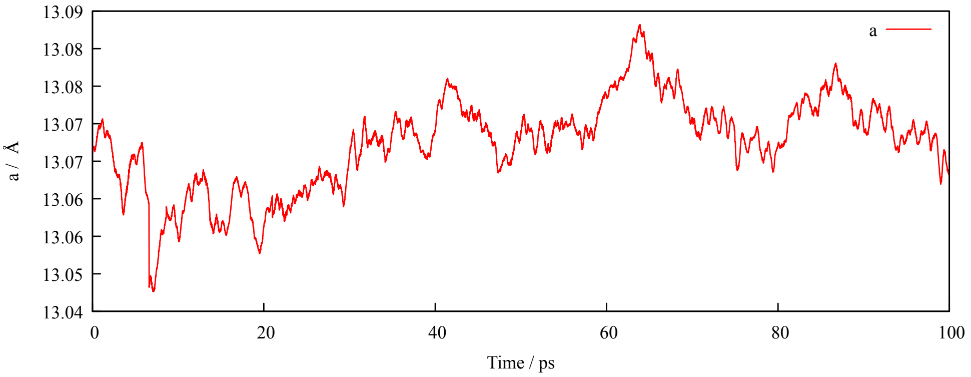

Figure S2: MOF5 cubic lattice constant a changes during the MD Simulation with respect to the k-point sampling (k=1)

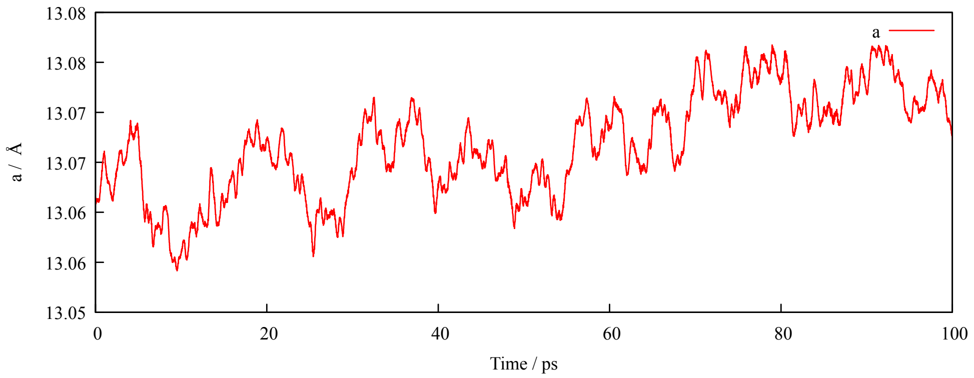

Figure S3: MOF5 cubic lattice constant a changes during the MD Simulation with respect to the k-point sampling (k=0)

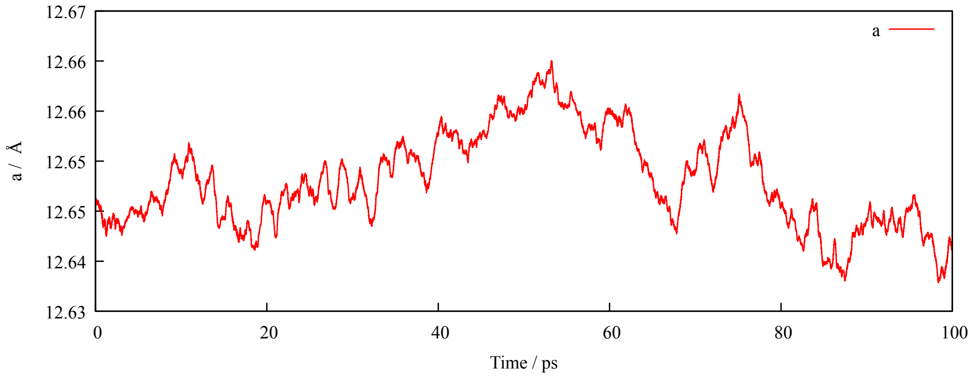

Figure S4: MOF5 cubic lattice constant a changes during the MD Simulation (xtb-GFN2) with respect to the k-point sampling (k=0)

S3.2 X-ray diffraction patterns

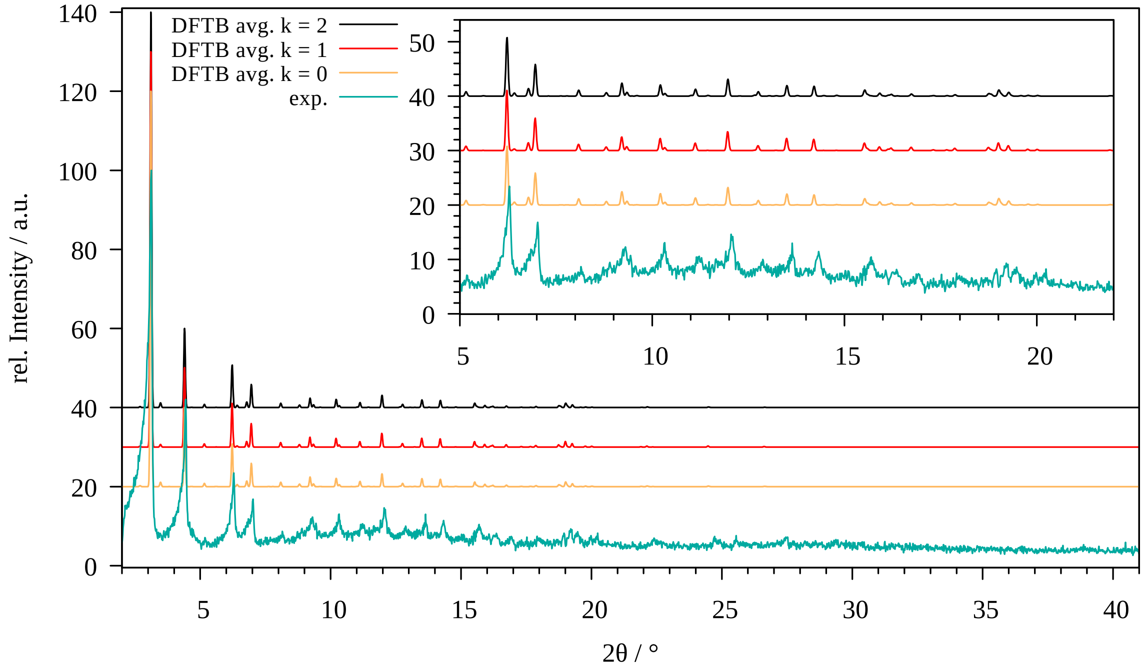

Figure S5: MOF-5 average X-ray diffraction patterns in dependence of the used k_{max} value

S4 DMOF

S4.1 Time evolution of lattice parameters

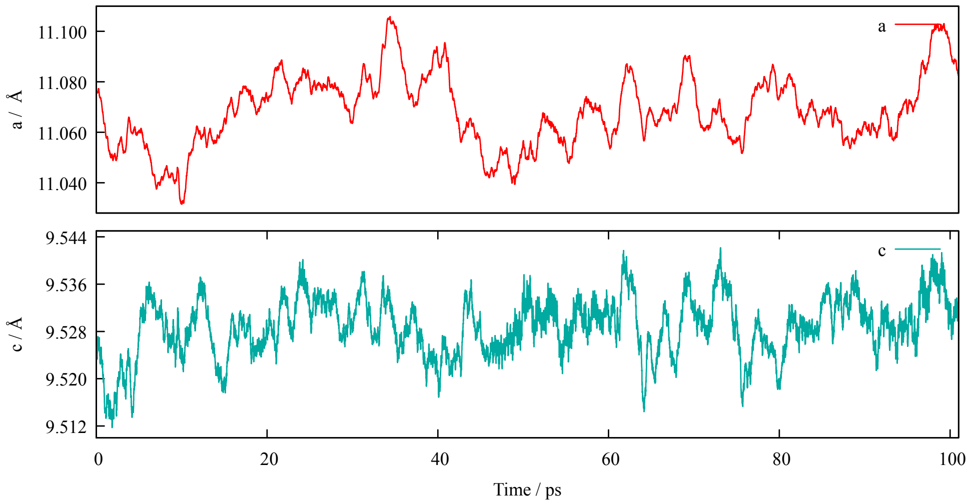

Figure S6: DMOF tetragonal lattice constants changes during the MD Simulation with k-point sampling (k=2)

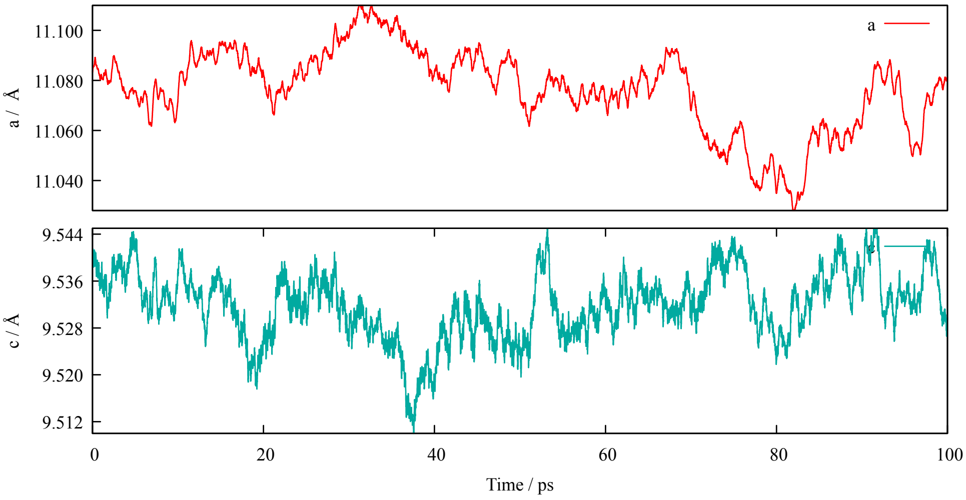

Figure S7: DMOF tetragonal lattice constants changes during the MD Simulation with k-point sampling (k=1)

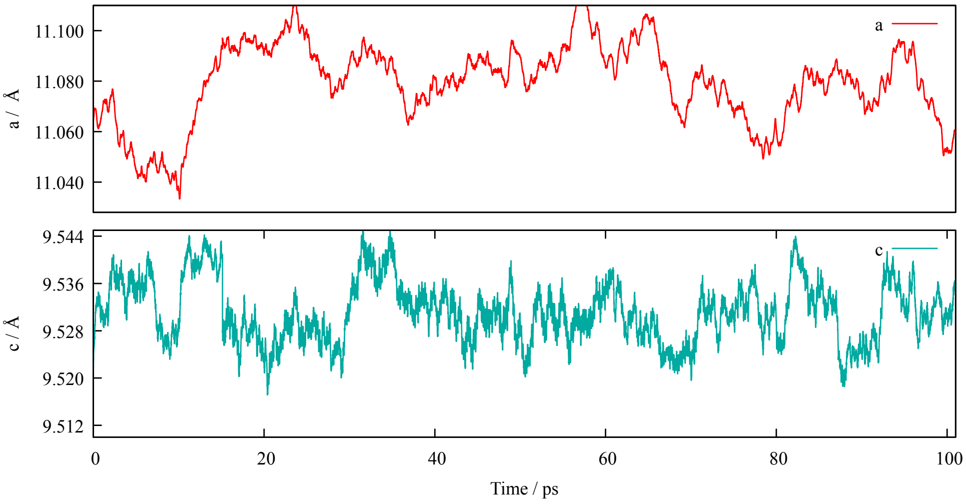

Figure S8: DMOF tetragonal lattice constants changes during the MD Simulation with k-point sampling (k=0)

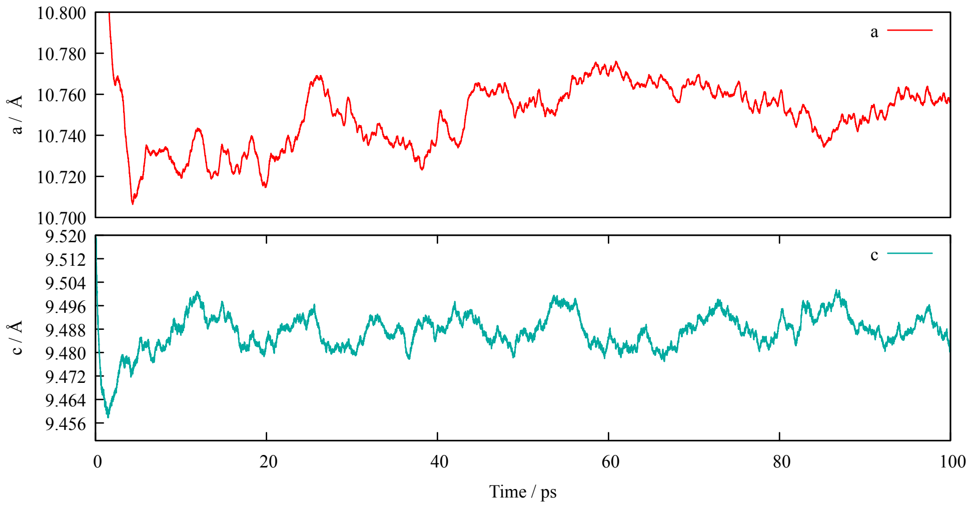

Figure S9: DMOF tetragonal lattice constants changes during the MD Simulation (xtb-GFN2) with k-point sampling (k=0)

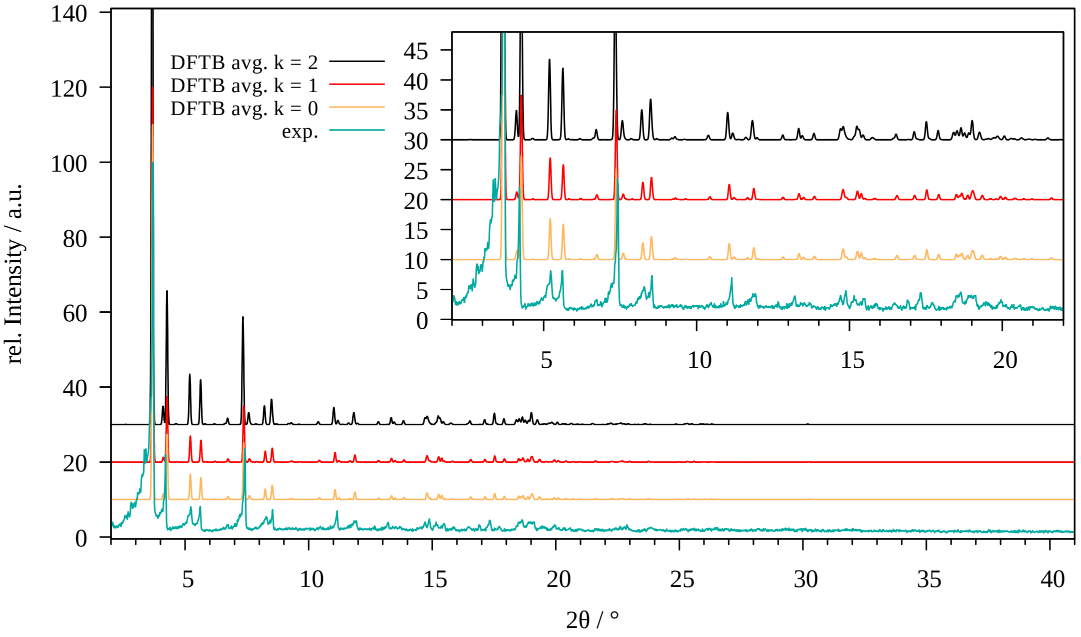

Figure S10: DMOF average X-ray diffraction patterns in dependence of the used k_{max} value

S5 ZnZn-RPM

S5.1 Time evolution of lattice parameters

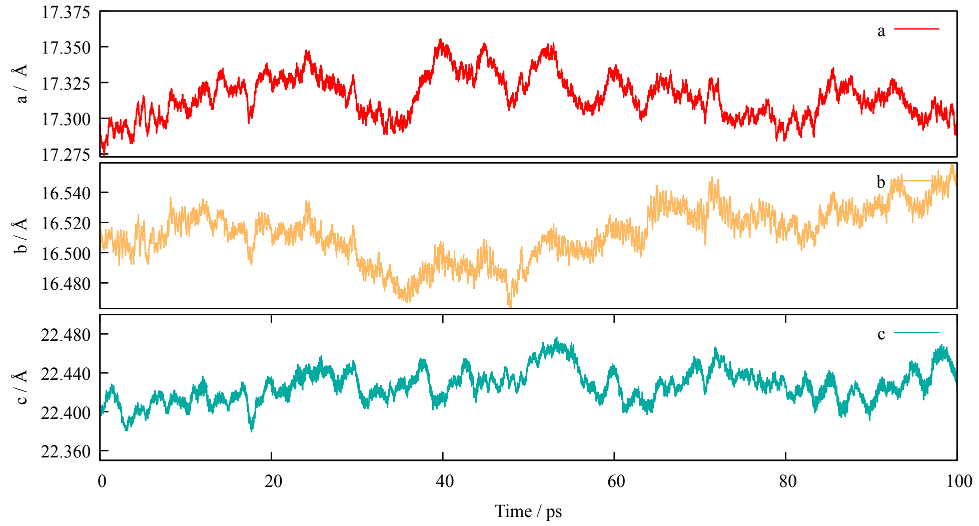

Figure S11: ZnZn-RPM anisotropic orthorombic lattice constants changes during the MD Simulation with k-point sampling (k=2)

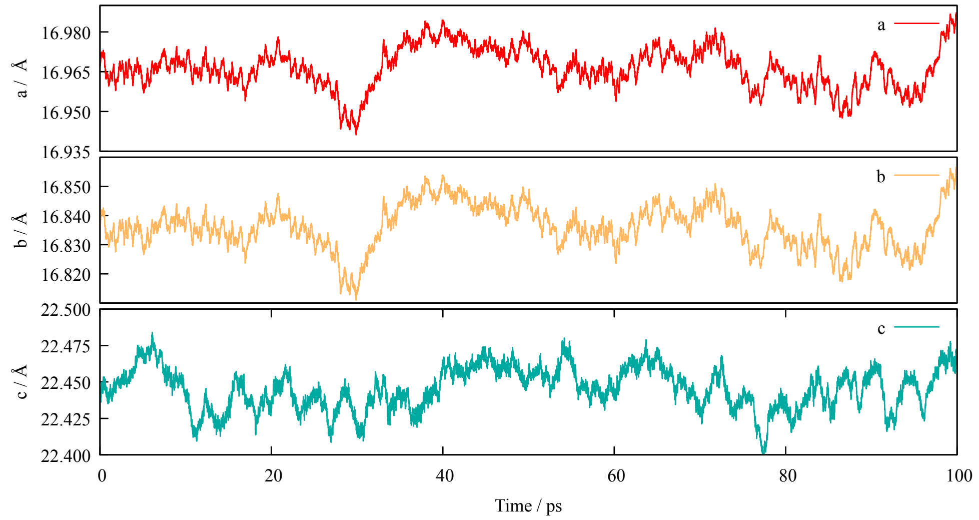

Figure S12: ZnZn-RPM xy-isotropic orthorombic lattice constants changes during the MD Simulation with k-point sampling (k=2)

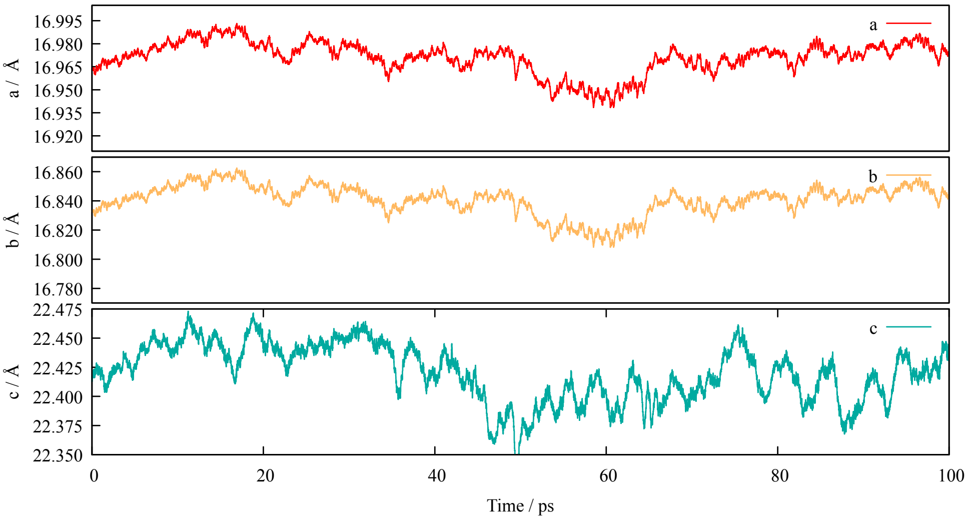

Figure S13: ZnZn-RPM xy-isotropic orthorombic lattice constants changes during the MD Simulation with k-point sampling (k=2)

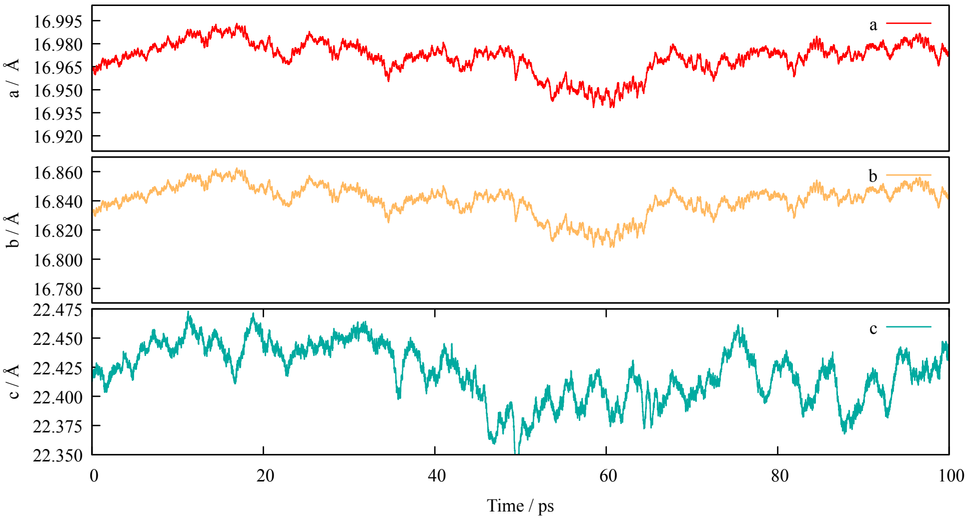

Figure S14: ZnZn-RPM xy-isotropic orthorombic lattice constants changes during the MD Simulation with k-point sampling (k=1)

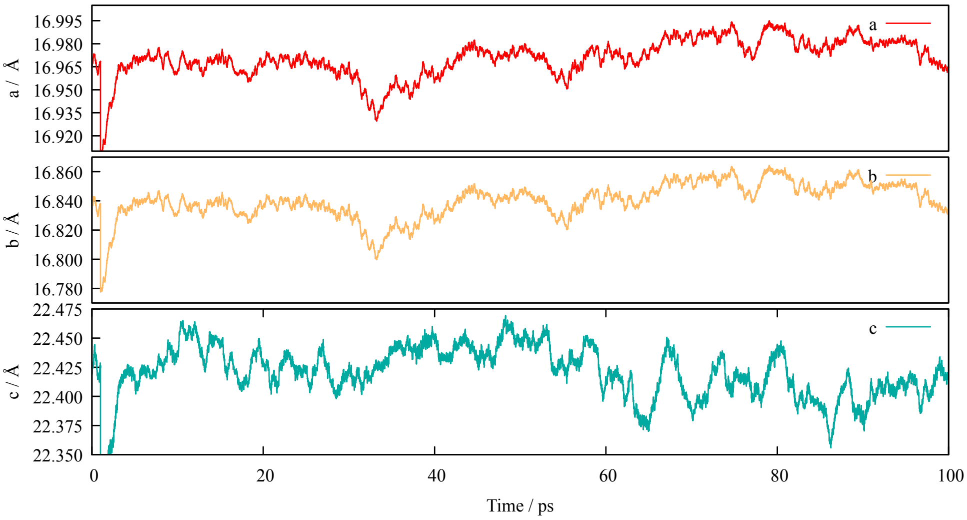

Figure S15: ZnZn-RPM xy-isotropic orthorombic lattice constants changes during the MD Simulation with k-point sampling (k=0)

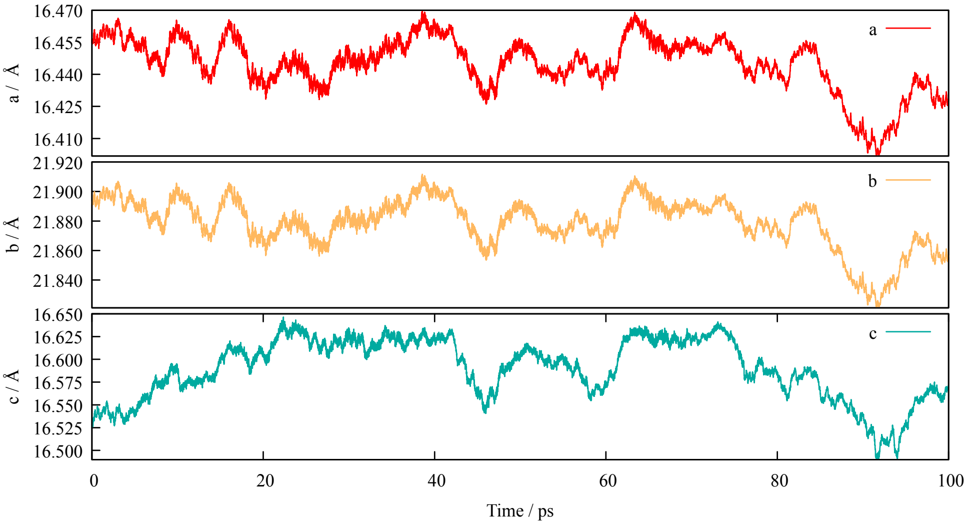

Figure S16: ZnZn-RPM xy-isotropic orthorombic lattice constants changes during the MD Simulation with k-point sampling (k=0)

S5.2 X-ray diffraction patterns

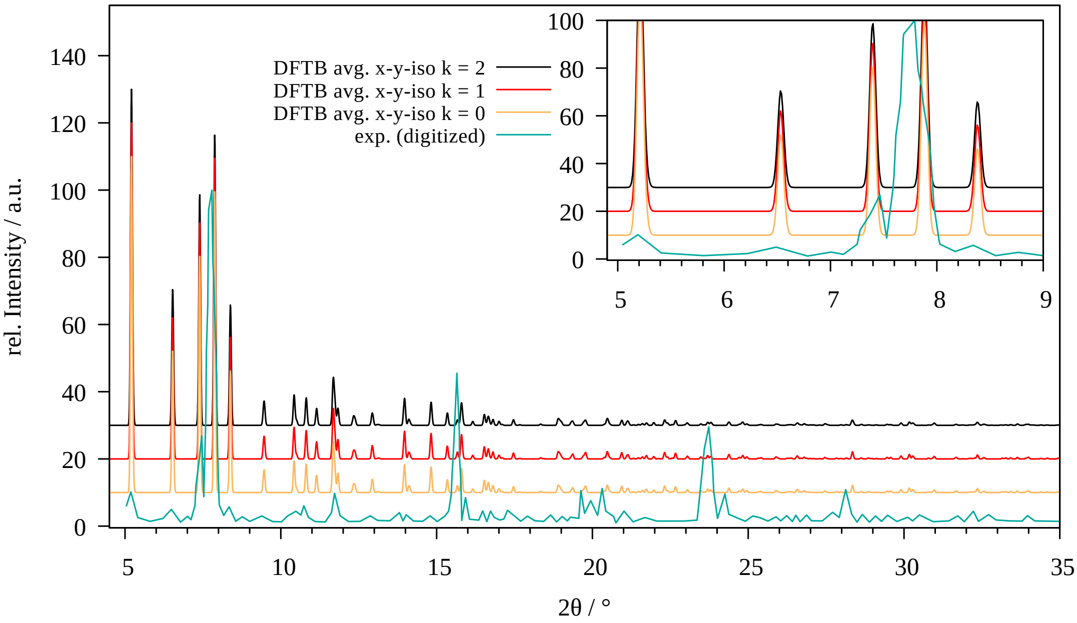

Figure S17: ZnZn-RPM average X-ray diffraction patterns in dependence of the used k_{max} value

S6 ZIF-8

S6.1 Time evolution of lattice parameters

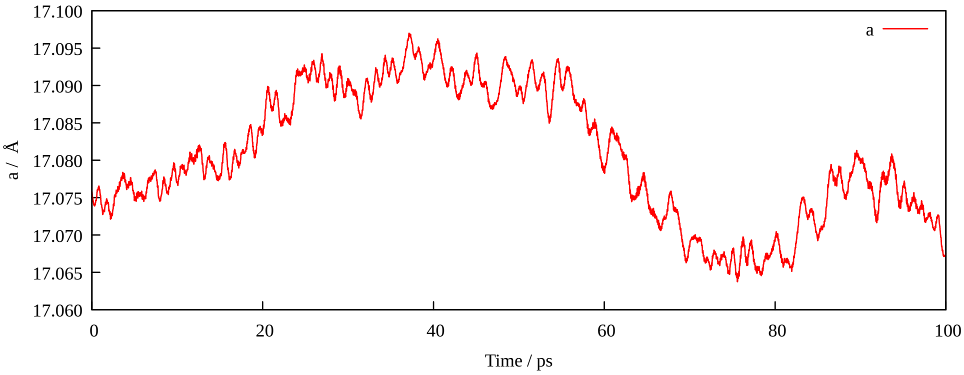

Figure S18: ZIF-8 cubic lattice constant a changes during the MD Simulation with respect to the k-point sampling (k=2)

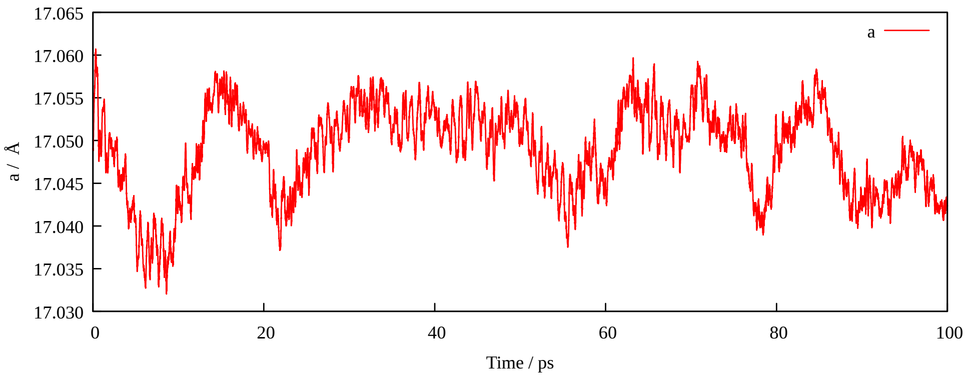

Figure S19: ZIF-8 cubic lattice constant a changes during the MD Simulation with respect to the k-point sampling (k=1)

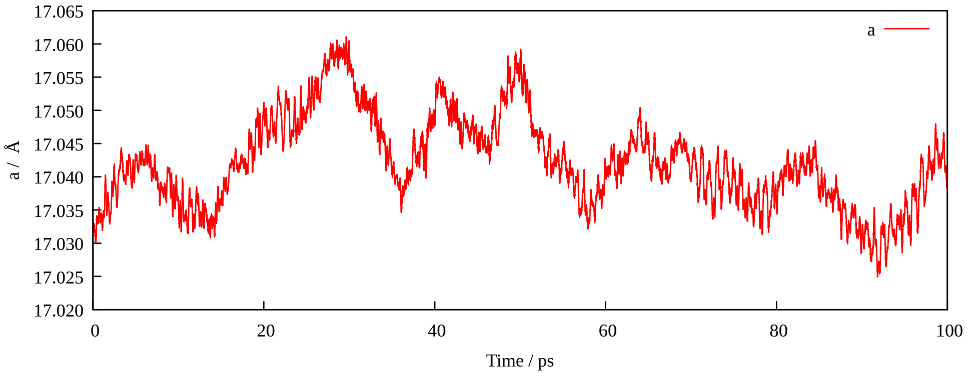

Figure S20: ZIF-8 cubic lattice constant a changes during the MD Simulation with respect to the k-point sampling (k=0)

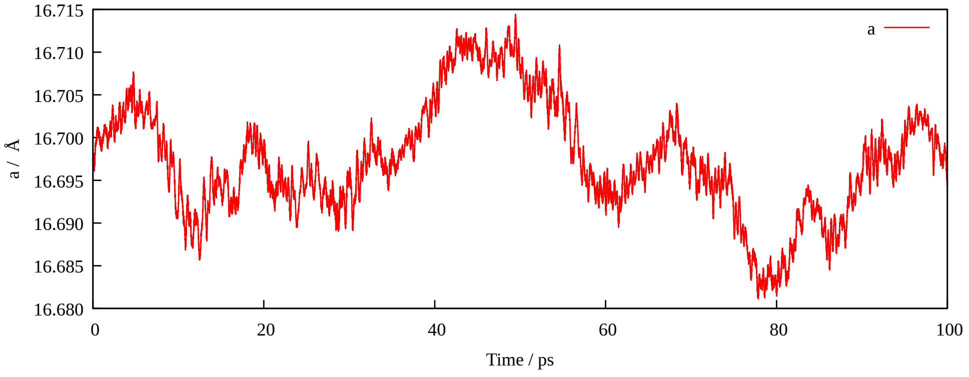

Figure S21: ZIF-8 cubic lattice constant a changes during the MD Simulation (xtb-GFN2) with respect to the k-point sampling (k=0)

S6.2 X-ray diffraction patterns

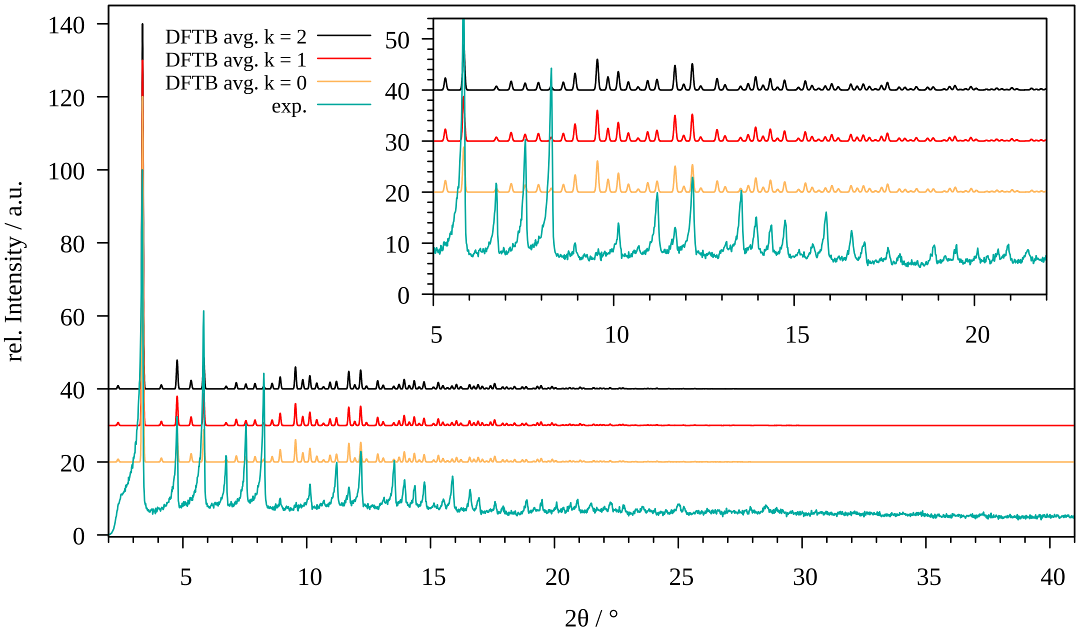

Figure S22: ZIF-8 average X-ray diffraction patterns in dependence of the used k_{max} value

S7 MIL-68(Al)

S8 Time evolution of lattice parameters

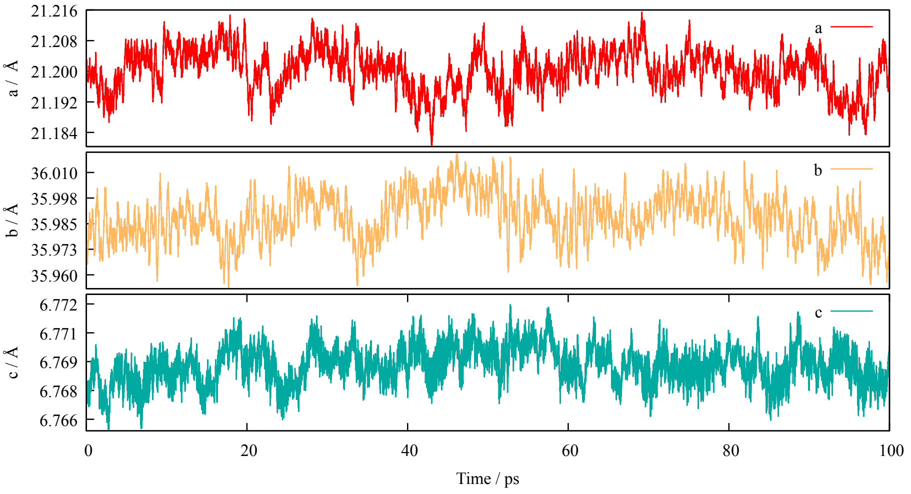

Figure S23: MIL-68(Al) orthorombic lattice constants changes during the MD Simulation with respect to the k-point sampling (k=1)

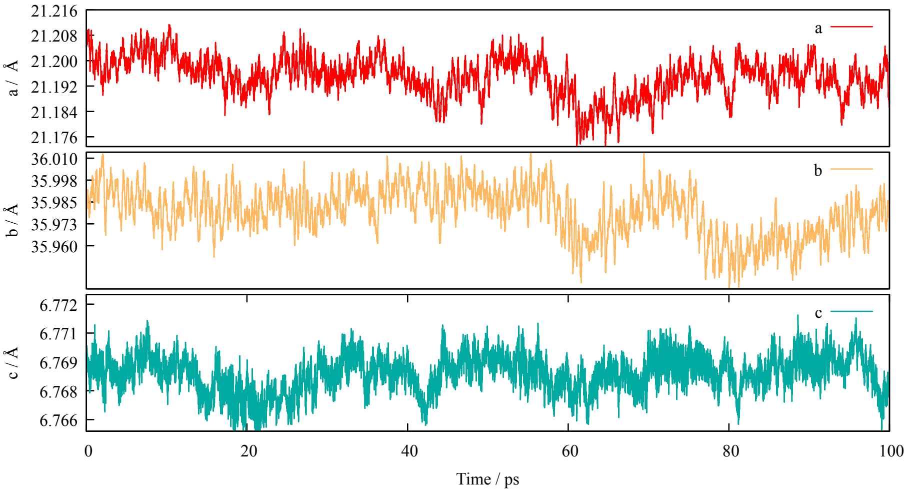

Figure S24: MIL-68(Al) orthorombic lattice constants changes during the MD Simulation with respect to the k-point sampling (k=0)

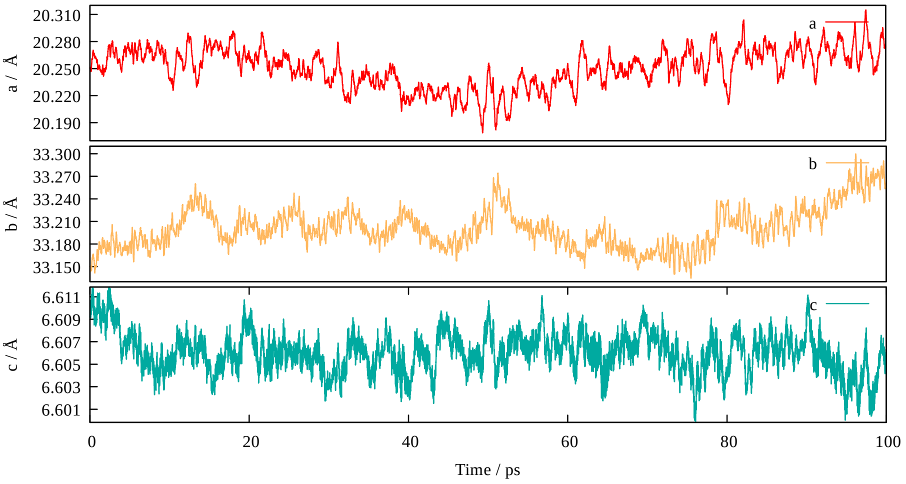

Figure S25: MIL-68(Al) orthorombic lattice constants changes during the MD Simulation (xtb-GFN2) with respect to the k-point sampling (k=1)

S8.1 X-ray diffraction patterns

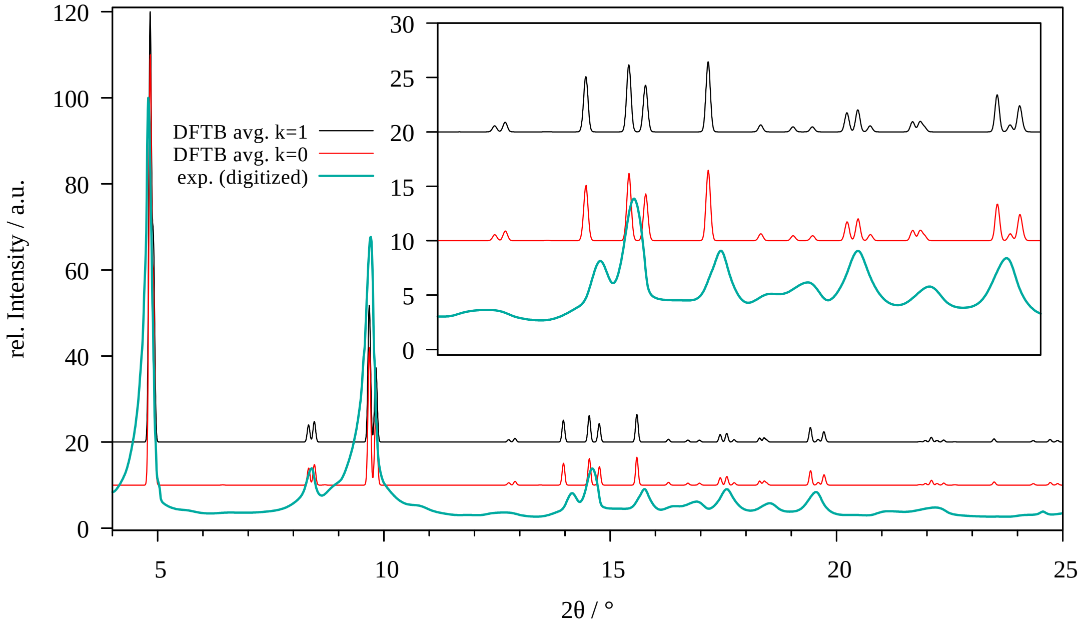

Figure S26: MIL-68(Al) average X-ray diffraction patterns in dependence of the used k_{max} value

S9 MIL-53(Al)

S10 Time evolution of lattice parameters

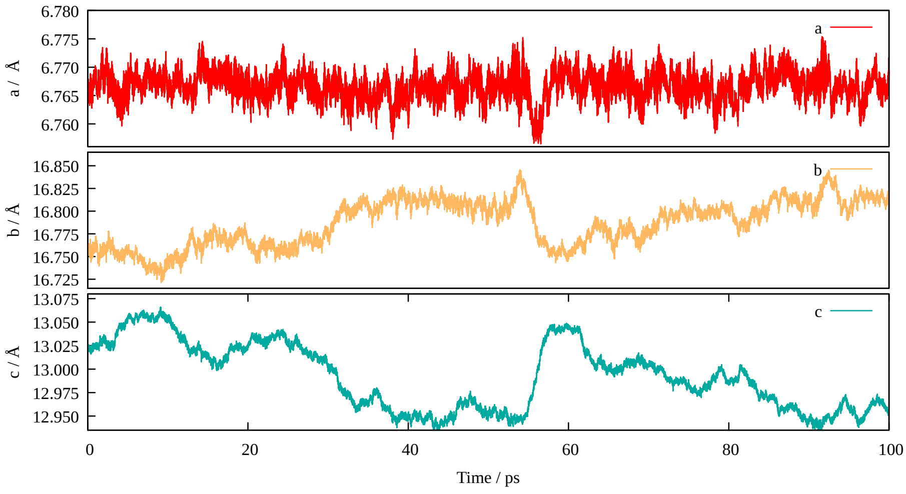

Figure S27: MIL-53(Al) orthorombic lattice constants changes during the MD Simulation with respect to the k-point sampling (k=1)

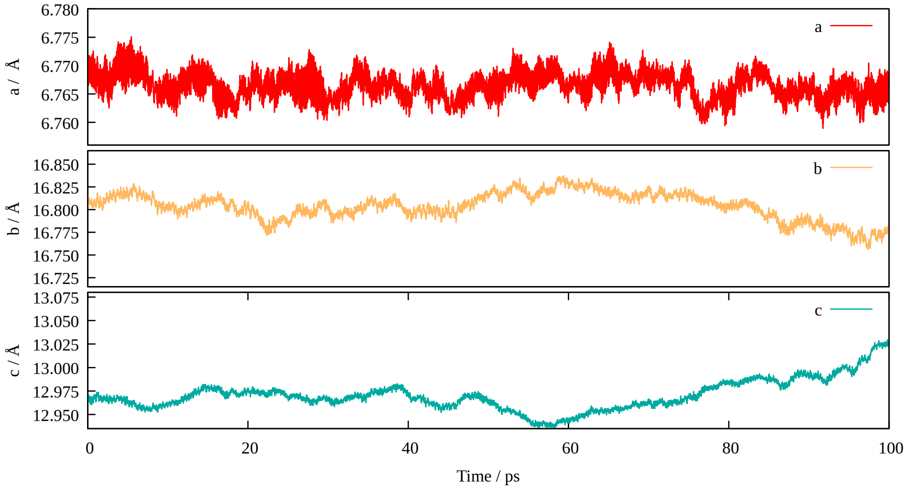

Figure S28: MIL-53(Al) orthorombic lattice constants changes during the MD Simulation with respect to the k-point sampling (k=0)

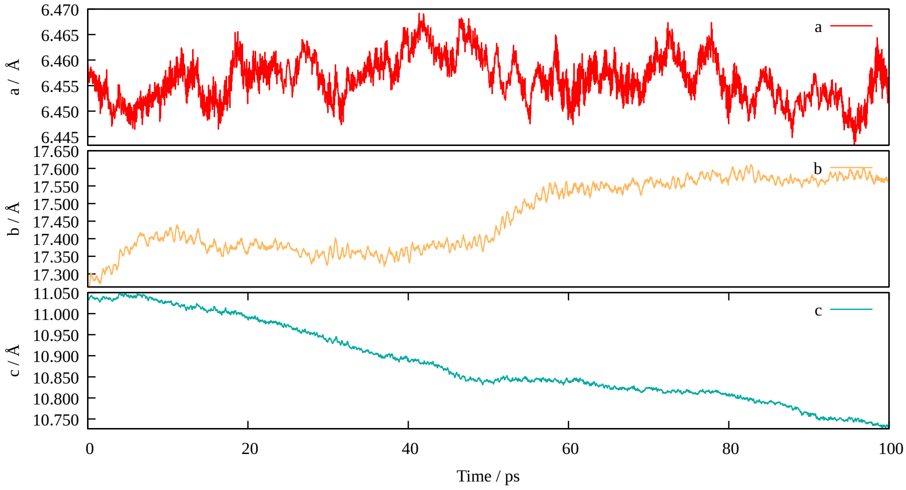

Figure S29: MIL-53(Al) orthorombic lattice constants changes during the MD Simulation (xtb-GFN2) with respect to the k-point sampling (k=1)

S10.1 X-ray diffraction patterns

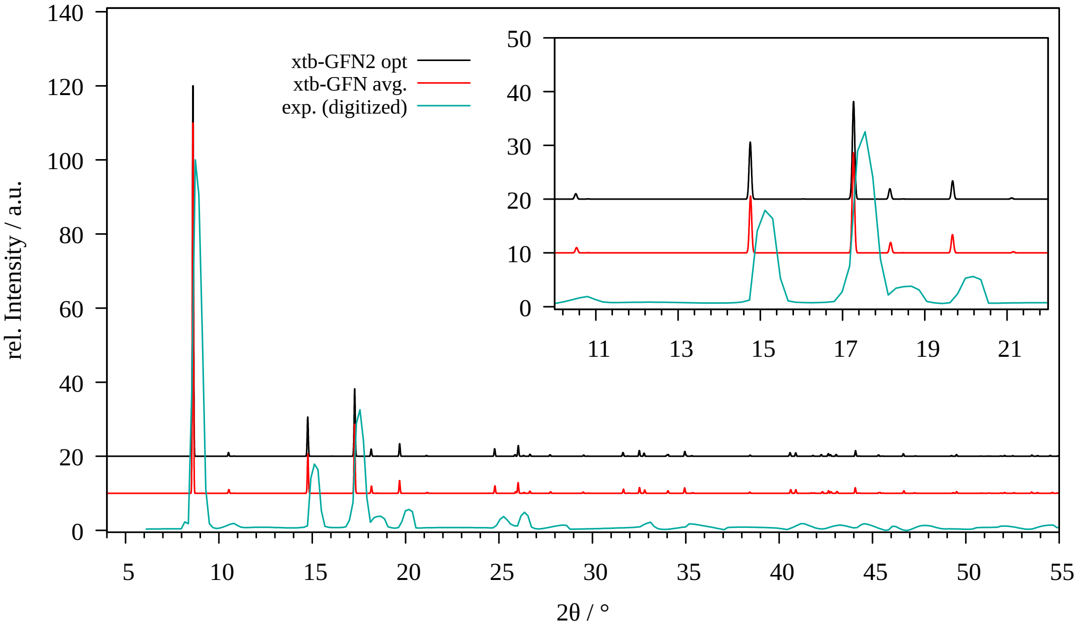

Figure S30: MIL-53(Al) average X-ray diffraction patterns in dependence of the used k_{max} value

S11 MIL-68(Ga)

S12 Time evolution of lattice parameters

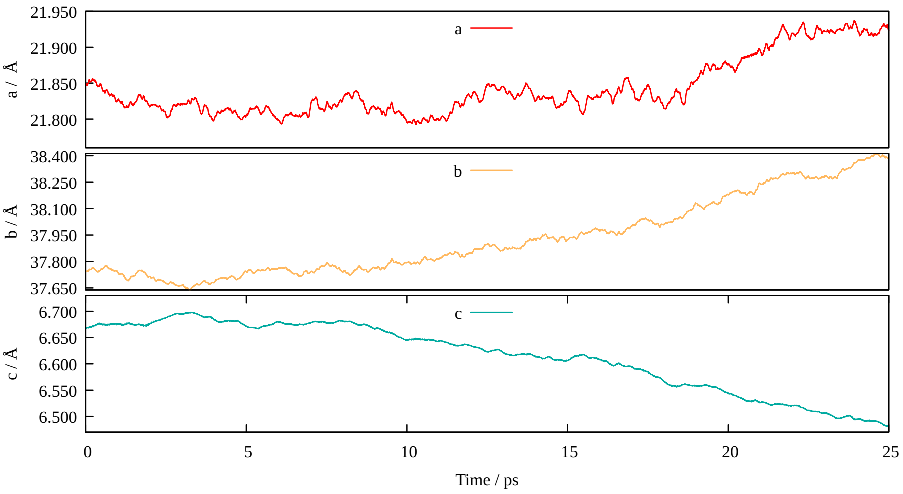

Figure S31: MIL-68(Ga) orthorombic lattice constants changes during the MD Simulation with respect to the k-point sampling (k=1)

References

(1) Rode, B. M.; Hofer, T. S.; Randolf, B. R.; Schwenk, C.; Xenides, D.; Vchirawongkwin, V. Ab initio quantum mechanical charge field (QMCF) molecular dynamics: a QM/MM – MD procedure for accurate simulations of ions and complexes. *Theor. Chem. Acc.* **2006**, *115*, 77.

(2) Weiss, A. K. H.; Hofer, T. S. Exploiting the Capabilities of Quantum Chemical Simulations to Characterise the Hydration of Molecular Compounds. *RSC Adv..* **2014**, *3*, 1606.

(3) Hofer, T. S. Perspectives for hybrid ab initio/molecular mechanical simulations of solutions: From complex chemistry to proton-transfer reactions and interfaces. *Pure Appl. Chem.* **2014**, *86*, 1.

(4) Hofer, T. S.; Tirler, A. O. Combining 2d-Periodic Quantum Chemistry with Molecular Force Fields: A Novel QM/MM Procedure for the Treatment of Solid-State Surfaces and Interfaces. *J. Chem. Theor. Comput.* **2015**, *11*, 5873.

(5) Hourahine, B.; Aradi, B.; Frauenheim, T. DFTB+, a software package for efficient approximate density functional theory based atomistic simulations. *J. Chem. Phys.* **2020**, *152*, 124101.

(6) Gaus, M.; Goez, A.; Elstner, M. Parametrization and Benchmark of DFTB3 for Organic Molecules. *J. Comput. Chem.* **2013**, *9*, 338–354.

(7) Gaus, M.; Lu, X.; Elstner, M.; Cui, Q. Parameterization of DFTB3/3OB for Sulfur and Phosphorus for Chemical and Biological Applications. *J. Comput. Chem.* **2014**, *10*, 1518–1537.

(8) Lu, X.; Gaus, M.; Elstner, M.; Cui, Q. Parametrization of DFTB3/3OB for Magnesium and Zinc for Chemical and Biological Applications. *J. Phys. Chem. B* **2015**, *119*, 1062–1082.

(9) Niehaus, T.; Elstner, M.; Frauenheim, T.; Suhai, S. Application of an approximate density-functional method to sulfur containing compounds. *J. Mol. Struct. (Theochem)* **2001**, *541*, 185–194.

(10) Gaus, M.; Lu, X.; Elstner, M.; Cui, Q. Parametrization and Benchmark of DFTB3 for Organic Molecules. *J. Chem. Theory Comput.* **2014**, *10*, 1518.

(11) Lu, X.; Gaus, M.; Elstner, M.; Cui, Q. Parametrization of DFTB3/3OB for Magnesium and Zinc for Chemical and Biological Applications. *J. Phys. Chem. B* **2014**, *119*, 1062–1082.

(12) Frenzel, J.; Oliveira, A. F.; Duarte, H. A.; Heine, T.; Seifert, G. Structural and Electronic Properties of Bulk Gibbsite and Gibbsite Surfaces. *Z. Anorg. Allg. Chem.* **2005**, *631*, 1267–1271.

(13) Luschtinetz, R.; Oliveira, A. F.; Frenzel, J.; Joswig, J.-O.; Seifert, G.; Duarte, H. A. Adsorption of phosphonic and ethylphosphonic acid on aluminum oxide surfaces. *Surf. Sci.* **2008**, *602*, 1347–1359.

(14) Monkhorst, H. J.; Pack, J. D. Special points for Brillouin-zone integrations. *Phys. Rev. B* **1976**, *13*, 5188–5192.

(15) Bannwarth, C.; Caldeweyher, E.; Ehlert, S.; Hansen, A.; Pracht, P.; Seibert, J.; Spicher, S.; Grimme, S. Extended tight-binding quantum chemistry methods. *WIREs Comput Mol Sci.* **2020**, *11*.

(16) Bannwarth, C.; Ehlert, S.; Grimme, S. GFN2-xTB—An Accurate and Broadly Parametrized Self-Consistent Tight-Binding Quantum Chemical Method with Multipole Electrostatics and Density-Dependent Dispersion Contributions. *J. Chem. Theory Comput.* **2019**, *15*, 1652–1671.

(17) Verlet, L. Computer "Experiments" on Classical Fluids. I. Thermodynamical Properties of Lennard-Jones Molecules. *Phys. Rev.* **1967**, *159*, 98–103.

(18) Swope, W. C.; Andersen, H. C.; Berens, P. H.; Wilson, K. R. A computer simulation method for the calculation of equilibrium constants for the formation of physical clusters of molecules: Application to small water clusters. *J. Chem. Phys.* **1982**, *76*, 637–649.

(19) Ryckaert, J.-P.; Ciccotti, G.; Berendsen, H. J. Numerical integration of the cartesian equations of motion of a system with constraints: molecular dynamics of n-alkanes. *J. Comput. Phys.* **1977**, *23*, 327–341.

(20) Andersen, H. C. Rattle: A "velocity" version of the shake algorithm for molecular dynamics calculations. *J. Comput. Phys.* **1983**, *52*, 24–34.

(21) Berendsen, H. J. C.; Postma, J. P. M.; van Gunsteren, W. F.; DiNola, A.; Haak, J. R. Molecular dynamics with coupling to an external bath. *J. Chem. Phys.* **1984**, *81*, 3684–3690.

(22) Farha, O. K.; Shultz, A. M.; Sarjeant, A. A.; Nguyen, S. T.; Hupp, J. T. Active-Site-Accessible, Porphyrinic Metal-Organic Framework Materials. *J. Am. Chem. Soc.* **2011**, *133*, 5652–5655.

(23) Rödl, M.; Reka, A.; Panic, M.; Fischereder, A.; Oberlechner, M.; Mairegger, T.; Kopacka, H.; Huppertz, H.; Hofer, T. S.; Schwartz, H. A. Fundamental Study of the Optical and Vibrational Properties of Fx-AZB@MOF systems as Functions of Dye Substitution and the Loading Amount. *Langmuir* **2022**, *38*, 4295–4309.

(24) Ashling, C. W.; Johnstone, D. N.; Widmer, R. N.; Hou, J.; Collins, S. M.; Sapnik, A. F.; Bumstead, A. M.; Midgley, P. A.; Chater, P. A.; Keen, D. A. et al. Synthesis and Properties of a Compositional Series of MIL-53(Al) Metal–Organic Framework Crystal-Glass Composites. *J. Am. Chem. Soc.* **2019**, *141*, 15641–15648.

(25) Tranchemontagne, D. J.; Hunt, J. R.; Yaghi, O. M. Room temperature synthesis of metal-organic frameworks: MOF-5, MOF-74, MOF-177, MOF-199, and IRMOF-0. *Tetrahedron* **2008**, *64*, 8553–8557.

(26) Dybtsev, D. N.; Chun, H.; Kim, K. Rigid and Flexible: A Highly Porous Metal–Organic Framework with Unusual Guest-Dependent Dynamic Behavior. *Angew. Chem. Int. Ed.* **2004**, *43*, 5033–5036.

(27) Embrechts, H.; Kriesten, M.; Ermer, M.; Peukert, W.; Hartmann, M.; Distaso, M. In situ Raman and FTIR spectroscopic study on the formation of the isomers MIL-68(Al) and MIL-53(Al). *RSC Adv.* **2020**, *10*, 7336–7348.
